# Integrating multi-receptor and neuron–glia mechanisms of ketamine's antidepressant action: a convergence framework for understanding efficacy

**DOI:** 10.3389/fnmol.2026.1950244

**Published:** 2026-10-21

**Authors:** Zuzanna Sulej, Magdalena Zielińska

**Affiliations:** 1 Department of Neurotoxicology, Mossakowski Medical Research Institute, Polish Academy of Sciences, Warsaw, Poland; 2 Doctoral School of Translational Medicine, Centre of Postgraduate Medical Education, Warsaw, Poland

**Keywords:** glia, ketamine, neurons, NMDA receptor, opioid receptors, psychiatric diseases

## Abstract

Ketamine has revolutionized psych iatric treatment, challenging monoamine-based paradigms through its capacity for rapid antidepressant action. While N-methyl-D-aspartate receptor (NMDAR) antagonism remains the canonical mechanism, this integrative review proposes a multi-receptor, neuron–glia convergence model to help account for ketamine's therapeutic efficacy. We outline the well-established disinhibition cascade, whereby preferential NMDAR blockade on GABAergic interneurons induces cortical disinhibition, triggering a transient glutamate surge and subsequent α-amino-3-hydroxy-5-methyl-4-isoxazolepropionic acid (AMPA) receptor activation. Furthermore, we discuss emerging, albeit indirect reports suggesting that ketamine and its metabolites may interact with the tropomyosin receptor kinase B (TrkB) transmembrane domain and modulate TrkB-related signaling. Beyond neurons, we consider the contributions of glial cells, particularly astrocytes, microglia, and oligodendrocyte lineage cells, as players in ketamine response in antidepressant effects. We further discuss evidence for opioid context-dependent effects that, at present, are insufficient to account for ketamine's broader antidepressant effects. Finally, we highlighted the sigma-1 receptor (S1R) as a chaperone modulator of ketamine-induced sustained neuroplasticity and intercellular neuron-glia communication. Collectively, the available data suggest that ketamine's efficacy likely arises from convergent actions across multiple molecular targets and both neuronal and glial cell types. Importantly, many components of this model remain hypothetical and require rigorous *in vivo* functional verification. By delineating testable mechanisms and distinguishing putative pro-plasticity effects from dissociative liabilities, this framework may inform precision psychiatry, biomarker-guided patient stratification, and the rational design of safer, next-generation rapid-acting antidepressants.

## 1 Clinical overview

### 1.1 Epidemiology

Psychiatric diseases, including depressive disorders [e.g., major depressive disorder (MDD), schizophrenia, bipolar disorder (BP)] and anxiety disorders, are the leading cause of disability worldwide, affecting approximately 5%−7% of the global population, with rates exceeding 10% in certain regions, according to the World Health Organization (Bromet et al., 2011; Vos et al., 2015; Ormel et al., 2022; Wolitzky-Taylor et al., 2026). The global burden of mental diseases has increased substantially, with the COVID-19 pandemic exacerbating prevalence rates across all age groups (Kleine et al., 2023). Depression accounts for significant disability-adjusted life years (DALYs), with estimates indicating that this group of diseases contributes to over 50 million DALYs globally, making it one of the top contributors to years lived with disability (Santomauro et al., 2026). The burden varies across the lifespan, with adolescents and young adults (ages 10–24) experiencing particularly steep increases in depression prevalence and DALYs (Yang et al., 2024; Zhu et al., 2025). Chronic depression carries higher disability weights than acute episodes, reflecting cumulative functional deterioration and treatment resistance (Kivimäki et al., 2023). Since women have approximately two times higher prevalence of mental disease, and men have higher suicide completion rates, the observed significant sex dimorphism adds clinical relevance and biological complexity (Otten et al., 2021).

The economic burden is equally staggering. Functional impairment extends beyond clinical symptoms to encompass reduced work productivity, social withdrawal, and increased mortality risk through suicide, which accounts for approximately 800,000 deaths annually worldwide. In the United States alone, the annual cost of mental disorders exceeds $210 billion when accounting for direct healthcare expenditures, workplace productivity losses, and suicide-related mortality (Greenberg et al., 2021; Lerner et al., 2021). In 2022, mental and behavioral disorders accounted for nearly 210,800 deaths across the EU, constituting 4.1% of all-cause mortality, with an age-adjusted rate of 42 per 100,000 population. The burden of these conditions was markedly age-dependent: standardized mortality rates among individuals aged ≥65 years exceeded those of younger populations by a factor of 45, substantially greater than the 22-fold differential observed for all-cause mortality. These data highlight the disproportionate impact of psychiatric and neurobehavioral conditions on elderly populations and underscore the need for targeted preventive and therapeutic interventions in this demographic (Eurostat, 2022).

### 1.2 Heterogeneity of depression disorders: subtypes and endophenotypes

Depression disorders represent a spectrum of syndromes with distinct inflammatory, metabolic, and glutamatergic signatures that may differentially respond to therapies (Fried and Nesse, 2015a,b).

Clinical heterogeneity manifests across multiple dimensions. The clinical subtypes briefly described below likely reflect distinct underlying pathophysiology with overlapping but differentiable mechanisms (Hasler et al., 2004; Nemeroff and Vale, 2005). The recognition that depression is not a single disease but a collection of biologically distinct syndromes has profound implications for clinical practice and research. Biomarker-guided treatment selection, using inflammatory markers, genetic testing, and eventually neuroimaging, represents the future of precision psychiatry, with the potential to reduce trial-and-error prescribing and improve outcomes for patients with this heterogeneous condition (Insel, 2014; Kuzminskaite et al., 2022).

*Melancholic depression* is characterized by profound anhedonia, psychomotor retardation, early morning awakening, and diurnal mood variation (Fink and Taylor, 2007; Martino et al., 2019). This subtype shows robust hypothalamic-pituitary-adrenal (HPA) axis hyperactivity (Lamers et al., 2013; Penninx et al., 2013) and may respond preferentially to tricyclic antidepressants (TCAs) (Valerio et al., 2018). Neuroimaging reveals hippocampal volume reduction and prefrontal cortical atrophy (Arnone et al., 2012; ENIGMA-Major Depressive Disorder Working Group et al., 2016). Melancholic depression responds preferentially to tricyclic antidepressants (TCAs) and electroconvulsive therapy (ECT), with response rates of approximately 50% for TCAs compared to 30% for SSRIs in meta-analytic data (Joyce et al., 2003; Valerio et al., 2018), while ECT achieves response rates of 60%−80% in melancholic patients (Fink and Taylor, 2007).

*Atypical depression* despite its name, is relatively common and is characterized by mood reactivity (the ability to brighten in response to positive events), increased appetite or weight gain, hypersomnia, leaden paralysis (a heavy, leaden feeling in the limbs), and long-standing interpersonal rejection sensitivity features mood reactivity, increased appetite, hypersomnia, leaden paralysis, and interpersonal rejection sensitivity (Stewart et al., 2010). This subtype is associated with inflammatory markers and may benefit from monoamine oxidase inhibitors (MAOIs) (Lamers et al., 2013). Inflammatory depression may arise from chronic medical conditions, obesity, or autoimmune disorders and shows a preferential response to monoamine oxidase inhibitors (MAOIs) (Leucht et al., 2007; Berk et al., 2013). This subtype frequently shows metabolic dysregulation, including insulin resistance and dyslipidemia, linking it to cardiometabolic comorbidities (Lamers et al., 2013; Lasserre et al., 2014).

*Treatment-resistant depression* is defined by failure to respond to at least two adequate trials of antidepressants from different classes (Rush et al., 2006). Treatment-resistant depression affects approximately 30% of patients with MDD and is associated with distinct genetic signatures, including childhood-onset variants (Fabbri et al., 2020). TRD patients show altered glutamatergic neurotransmission and may be particularly responsive to ketamine (Lener et al., 2017).

*Inflammatory/immune-driven depression* represents a subtype characterized by elevated peripheral inflammatory markers [C-reactive protein (CRP), interleukin-6 (IL-6)], increased kynurenine pathway activation, and elevated brain glutamate levels measured by magnetic resonance spectroscopy (MRS) (Haroon et al., 2016; Raison et al., 2006). Inflammatory depression may arise from chronic medical conditions, obesity, or autoimmune disorders and shows a preferential response to anti-inflammatory augmentation strategies. Clinically, inflammatory depression often presents with prominent fatigue, psychomotor slowing, cognitive impairment (“brain fog”), and somatic symptoms (Dantzer et al., 2008).

*Stress-susceptible* vs. *stress-resilient* phenotypes emerge from gene-environment interactions. Individuals with so far identified high-risk genetic variants e.g., functional variants in FKBP5 (a regulator of glucocorticoid receptor sensitivity), the BDNF Val66Met polymorphism, or the short allele of the serotonin transporter-linked polymorphic region (5-HTTLPR) exposed to early-life adversity show heightened vulnerability to depression (Binder, 2008; Caspi et al., 2003; Heim and Binder, 2012; Klengel et al., 2013). Conversely, stress-resilient individuals maintain adaptive coping despite adversity, potentially through enhanced regulation of the prefrontal cortex (PFC) circuits and neuroplasticity (Russo et al., 2012).

The clinical subtypes likely reflect distinct underlying pathophysiologies, and identification of endophenotypes may serve as targets for personalized treatment approaches, including cognitive therapies and uncoupling patients from dopaminergic or glutamatergic interventions, likely to help identify individuals at risk for specific depression subtypes.

Importantly, in the narrative review by Fried (2026), a fundamental shift in understanding of mental health and diagnostic classification has been proposed. In the review, the current attempt at classification is centered on the Homeostatic Property Cluster (HPC) framework, which represents a future direction: instead of seeking rigid, fixed categories, it embraces the complexity and fluidity of mental health problems as clusters of interrelated properties that naturally overlap and vary. The proposed approach moves beyond traditional diagnostic labels by focusing on the underlying patterns and mechanisms connecting symptoms, traits, and biological, psychological, and social factors. Flexible and purpose-driven classifications allow for multiple coexisting frameworks tailored to different clinical, research, and policy needs. Ultimately, HPC strategy encourages personalized, dynamic, and theory-driven approaches to diagnosis and treatment, paving the way for more effective and nuanced mental health care in the future (Fried, 2026).

### 1.3 Clinical comorbidities

Depressive disorders frequently co-occur with comorbid psychiatric conditions. In a cohort study of 190 patients with MDD, 35.3% exhibited at least one psychiatric comorbidity, including anxiety disorders (21.1%), obsessive–compulsive disorder (OCD; 4.7%), post-traumatic stress disorder (PTSD; 4.2%), and alcohol dependence (0.5%) (Ittasakul et al., 2014). Notably, the comorbidity rate between depression and anxiety disorders has been reported to range from 47% to 58% in multiple studies (Brown et al., 2001; Lamers et al., 2011).

Anxiety disorders are characterized by excessive and persistent fear, worry, and avoidance that are disproportionate to actual threat. They include generalized anxiety disorder, panic disorder, social anxiety disorder, specific phobias, separation anxiety disorder, and agoraphobia (Penninx et al., 2021). Pathophysiological characteristics involve amygdala hyperreactivity, impaired prefrontal regulation, HPA-axis dysregulation, reduced hippocampal neuroplasticity, and disturbances in GABAergic, serotonergic, and noradrenergic neurotransmission (Craske and Stein, 2016).

PTSD, currently classified as a trauma- and stressor-related disorder, develops after traumatic exposure and is marked by intrusive re-experiencing, avoidance, negative mood, and hyperarousal (Penninx et al., 2021). It is associated with increased amygdala activity, prefrontal hypoactivity with impaired fear extinction, reduced hippocampal volume, altered noradrenergic and glutamatergic signaling, HPA-axis dysregulation, and trauma-related epigenetic changes (Beatty et al., 2026; Etkin and Wager, 2007).

OCD is characterized by obsessions and compulsions and is linked to dysfunction of cortico-striato-thalamo-cortical circuits, together with serotonergic, dopaminergic, and glutamatergic abnormalities and altered synaptic plasticity (Hinojosa et al., 2024; Menzies et al., 2008).

Depression may also arise secondary to chronic medical conditions, particularly chronic pain, where depressive symptoms occur in approximately 49.1% of patients (Annagür et al., 2014). Chronic pain is associated with maladaptive neuroplastic changes in the anterior cingulate cortex (ACC), insula, and prefrontal cortex (PFC), as well as glutamatergic and inflammatory dysregulation (Baliki and Apkarian, 2015; Sheng et al., 2017). Depression is also strongly associated with suicidality; about 19% of treatment-seeking chronically depressed individuals report at least one suicide attempt (Ernst et al., 2020).

### 1.4 Basis of pathomechanism: from monoamines to glutamate as evolving hypotheses

The monoamine hypothesis, which posits that depression results from deficiencies in serotonin (5-HT), norepinephrine (NE), and dopamine (DA), has dominated psychiatric research and pharmacotherapy for over five decades (Coppen, 1967; Schildkraut, 1965). Conventional antidepressants, including selective serotonin reuptake inhibitors (SSRIs), serotonin-norepinephrine reuptake inhibitors (SNRIs), and TCAs, target monoaminergic systems by increasing synaptic availability of these neurotransmitters (Belmaker and Agam, 2008). While effective for many patients, this paradigm faces significant limitations. Approximately 30%−40% of patients fail to achieve remission even after multiple adequate trials (treatment-resistant depression), and therapeutic effects typically require 4–8 weeks to emerge, a temporal profile inconsistent with the immediate pharmacological action of these agents (Rush et al., 2006; Trivedi et al., 2006).

The delayed onset of clinical improvement suggests that monoamine replenishment is necessary but insufficient; thus, emerging evidence now implicates glutamatergic dysfunction as a core pathophysiological mechanism, shifting the focus from neurotransmitter depletion to impaired synaptic plasticity and connectivity (Sanacora et al., 2008; Duman and Aghajanian, 2012; Popoli et al., 2012).

Glutamate is the brain's primary excitatory neurotransmitter, mediating fast synaptic transmission, synaptic plasticity, learning, and memory through activation of ionotropic receptors [N-methyl-D-aspartate (NMDA), α-amino-3-hydroxy-5-methyl-4-isoxazolepropionic acid (AMPA), kainate] and metabotropic glutamate receptors (mGluRs) (Ferraguti and Shigemoto, 2006; Niswender and Conn, 2010; Traynelis et al., 2010). In depression, glutamatergic dysfunction has emerged as a core pathophysiological mechanism, supported by converging evidence from neuroimaging, molecular biology, and clinical trials (Moriguchi et al., 2019; McGrath et al., 2022).

The discovery that ketamine, an uncompetitive NMDA receptor antagonist, produces a rapid and robust antidepressant effect has fundamentally challenged traditional models of depression pathophysiology and treatment. Unlike conventional antidepressants that require weeks to months for therapeutic effects, ketamine can produce significant symptom reduction within hours, with effects persisting for days to weeks following a single administration (Krystal et al., 2019; Chen et al., 2024).

The mechanisms underlying rapid antidepressant actions of ketamine have been the subject of intensive investigation over the past two decades (Pittenger and Duman, 2008; Zanos et al., 2018; Zanos and Gould, 2018). While initial mechanistic models focused primarily on glutamatergic neurotransmission through NMDA receptor antagonism, influencing synaptic plasticity and neurotransmitter release, accumulating evidence reveals a far more complex picture, yet interconnected molecular mechanisms that involve multiple neurotransmitter systems, diverse receptor targets, and cell-type-specific effects (Krystal et al., 2013; Carboni et al., 2021; Ma et al., 2022). Of particular interest in therapeutic effects of ketamine is the emerging role of sigma 1 receptors and opioid receptors, as was demonstrated by studies showing that the opioid receptor antagonist naltrexone (NTX) can attenuate ketamine's antidepressant and anti-suicidal actions (Williams et al., 2018, 2019).

## 2 Ketamine, a new antidepressant

Ketamine, originally developed as an anesthetic, has emerged as the most significant breakthrough in depression treatment in over 50 years. In 2019, the FDA approved esketamine (S-ketamine) nasal spray for treatment-resistant depression, marking the first approval of a rapid-acting antidepressant with a novel mechanism of action (Kryst et al., 2020b; McIntyre and Jain, 2024). Three forms of ketamine have been investigated: racemic ketamine, S-ketamine (esketamine), and R-ketamine (arketamine). Figure 1 summarizes the enantiomers' key pharmacological characteristics (Yang et al., 2015; Popova et al., 2019; Bonaventura et al., 2021; Jelen et al., 2021; Kalkman, 2023; Shafique et al., 2024).

**Figure 1:**
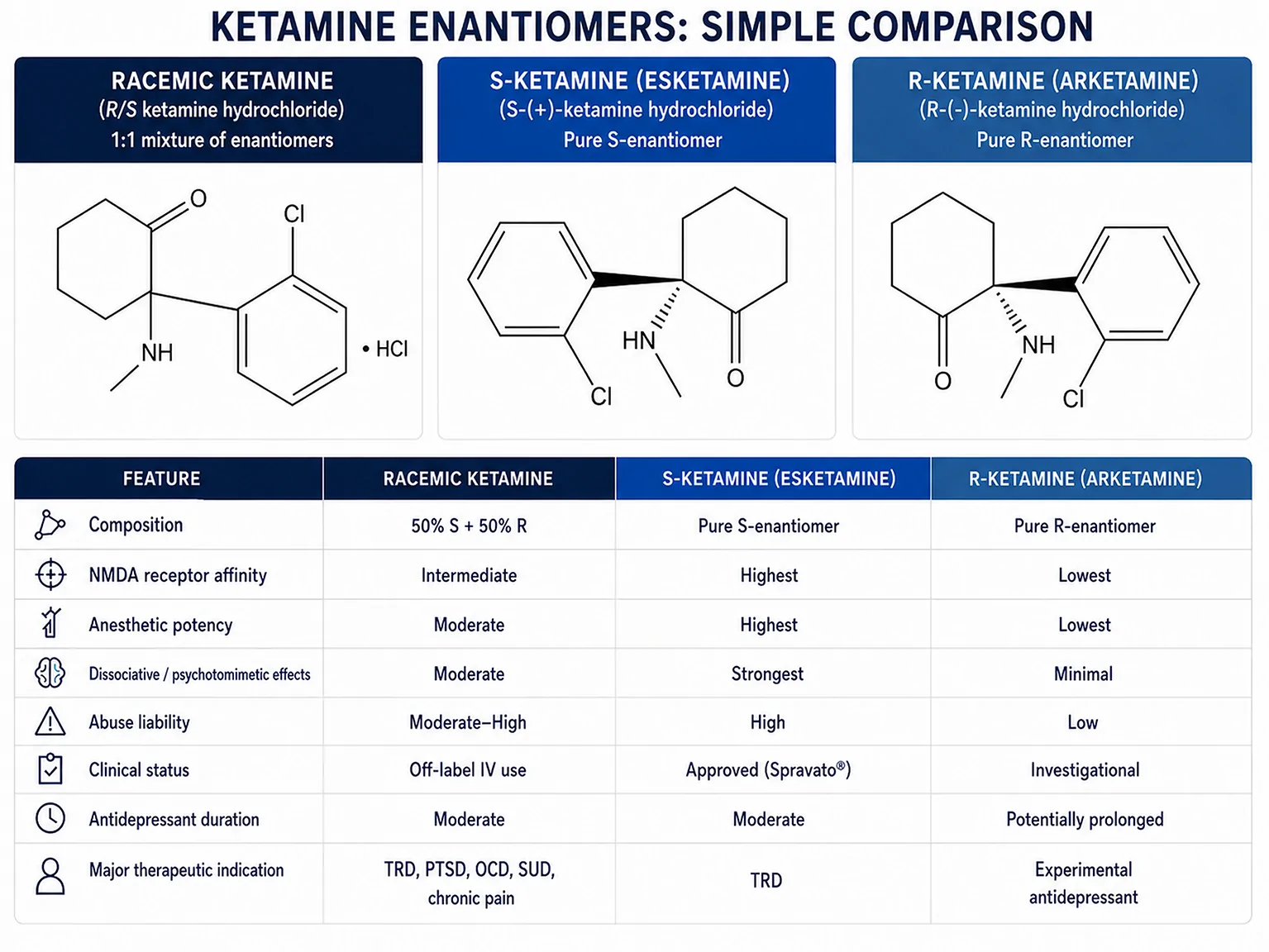
Racemic ketamine consists of a 1:1 mixture of S- and R-enantiomers, whereas esketamine and arketamine represent the isolated S- and R-enantiomers, respectively. Key differences include composition, NMDA receptor affinity, anesthetic potency, dissociative/psychotomimetic effects, abuse liability, clinical status, antidepressant duration, and major therapeutic indications; the comparison highlights that S-ketamine generally shows higher NMDA receptor affinity and anesthetic potency, while R-ketamine is associated with lower dissociative effects and is being investigated for its potential antidepressant properties.

Approximately 2,000 active or completed clinical trials related to ketamine and psychiatric disorders have been registered globally (Mandal et al., 2019; Kryst et al., 2020a,b; Yavi et al., 2022). Most of these studies focus on depression and its subtypes, particularly treatment-resistant depression. A significant number of trials also examine the therapeutic potential of ketamine in anxiety disorders, PTSD, OCD, bipolar disorder, chronic pain and physiological disorders, suicidal ideation, and substance use disorder (Wilkinson et al., 2018; Rodolico et al., 2024; Shen et al., 2024, 2025; Vekhova et al., 2025; Yin et al., 2026). To provide a broader overview of current clinical research on ketamine in psychiatry, selected ongoing clinical trials were summarized according to study population, sample size, year of initiation, and geographical distribution. This landscape highlights the main psychiatric indications currently investigated, including depression, anxiety disorders, PTSD, bipolar disorder, suicidal ideation, and OCD, and illustrates the international scope of ketamine-related clinical research (Figure 2). A complete table containing more detailed information on the selected clinical trials is provided in Supplementary Table S1.

**Figure 2:**
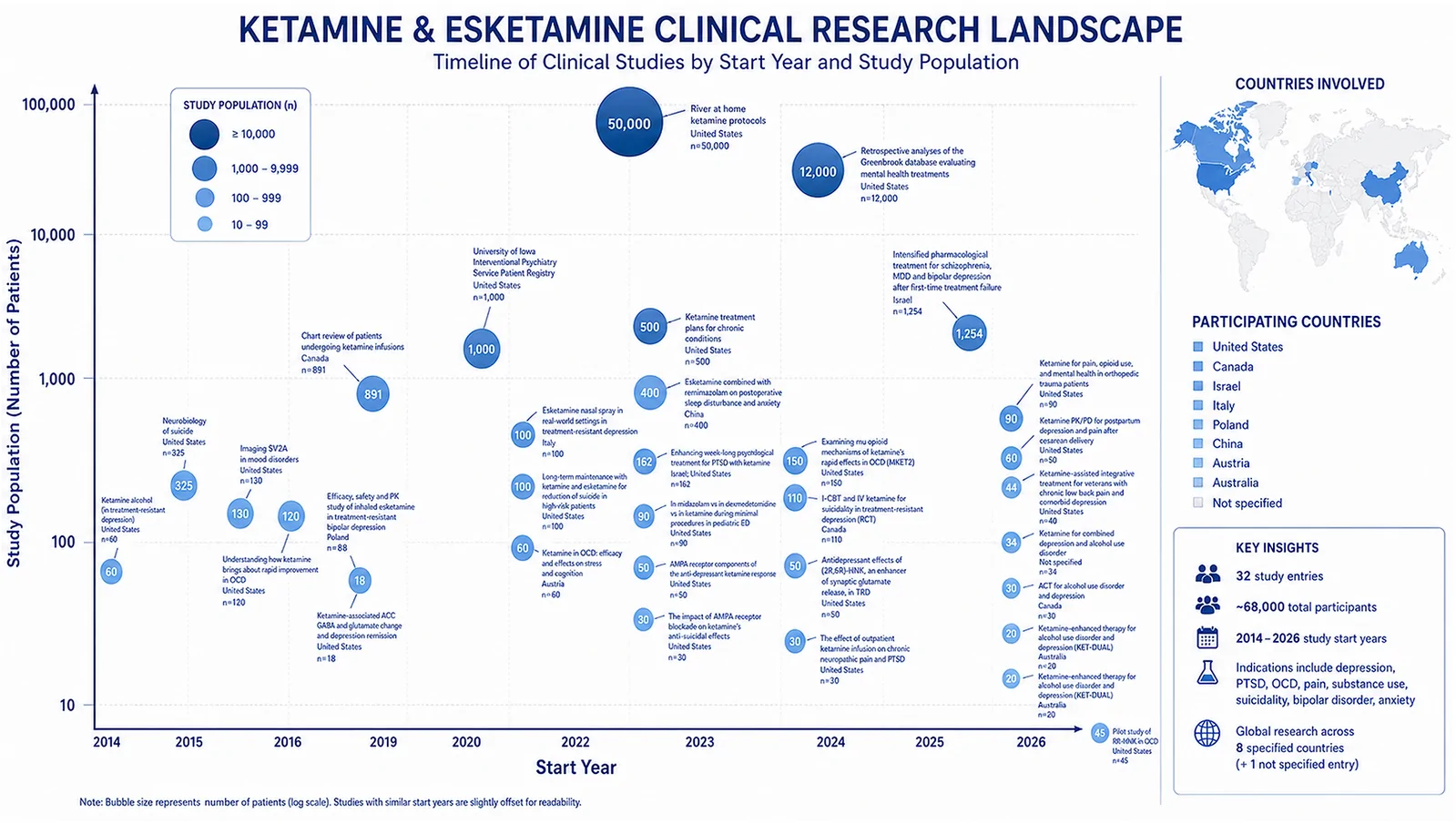
Selected clinical studies were included to represent major psychiatric indications, including depression, anxiety disorders, PTSD, bipolar disorder, suicidal ideation, and OCD. The figure focuses on currently active, large-enrollment, and internationally diverse trials in which ketamine is confirmed as a treatment arm. Studies are grouped according to the number of enrolled participants and arranged by trial start year, allowing visualization of the clinical research timeline and study population size. The accompanying map on the right highlights the countries in which the selected studies are conducted, while the mini legend summarizes the main indications and graphical coding used in the figure.

Although numerous clinical studies have investigated the effects of ketamine, its precise mechanism of action remains incompletely understood (Quintero et al., 2025). The most widely accepted mechanism involves antagonism of the N-methyl-D-aspartate receptor (NMDAR) and activation of the α-amino-3-hydroxy-5-methyl-4-isoxazolepropionic acid (AMPA) receptor (Gibuła-Tarłowska et al., 2025; Quintero et al., 2025). Additionally, evidence indicates that ketamine effects may be modulated by opioid receptors and the tropomyosin receptor kinase B (TrkB) signaling pathway (Levinstein et al., 2025a; Zhang et al., 2025).

In this review, we provide an integrative synthesis of current evidence on ketamine's mechanisms of action, with specific emphasis on: (1) receptor-specific contributions to antidepressant effects, (2) cell-type-specific mechanisms across neural cells, and (3) the integration of the opioidergic system in producing therapeutic outcomes. The following sections synthesize current knowledge on mechanisms critical for optimizing ketamine-based treatments and developing next-generation rapid-acting antidepressants with improved efficacy and safety profiles, starting with a brief description of ketamine and progressing toward an integrated, state-of-the-art perspective.

## 3 NMDA receptor and evidence for interaction with ketamine

### 3.1 Primary ketamine target: NMDA in neurons

The primary target of ketamine is the NMDAR, an ionotropic glutamate receptor for the principal excitatory neurotransmitter in the central nervous system (CNS), commonly associated with neuronal cells (Krystal et al., 2013).

NMDARs were firstly discovered in 1970s (Papouin and Oliet, 2017) and are encoded by seven genes from three related gene families that together determine receptor subunit composition: the GluN1 family—GRIN1, GluN2—GRIN2A, GRIN2B, GRIN2C, GRIN2D, and GluN3—GRIN3A, GRIN3B (Bai and Hoffman, 2009; Paoletti et al., 2013).

#### 3.1.1 Overview of NMDA receptors in neurons

Neuronal NMDARs are tetrameric receptors containing two obligatory GluN1 subunits that bind glycine or D-serine paired with two additional subunits: either glutamate-binding GluN2(A-D) subunits or, rarely, glycine-binding GluN3(A-B) (Paoletti et al., 2013; Hansen et al., 2018; Rajani et al., 2020). Each variable defines the biophysical, pharmacological, and signaling attributes of the NMDAR complex (Paoletti et al., 2013). GluN2A-containing receptors exhibit faster deactivation kinetics, higher open probability, and preferential coupling to pro-survival signaling pathways (ERK, CREB, BDNF transcription) (Hardingham and Bading, 2010; Zhang and Luo, 2013; Glasgow et al., 2015). GluN2B-containing receptors show slower deactivation and coupling to pro-apoptotic pathways (p38 MAPK, DAPK1, caspase activation) (Martel et al., 2012; Ge and Wang, 2023). GluN2C and GluN2D subunits are predominantly expressed in the cerebellum, thalamus, and interneurons, with lower calcium permeability and distinct pharmacological profiles (Paoletti et al., 2013). On the other hand, GluN3 subunits reduce calcium permeability and magnesium sensitivity, forming “excitatory glycine receptors” that may mediate neuroprotection (Grand et al., 2018; Kehoe et al., 2013). Neuronal NMDARs typically form GluN1/GluN2A or GluN1/GluN2B di-heteromers, or GluN1/GluN2A/GluN2B tri-heteromers (Paoletti et al., 2013).

Classical NMDAR activation requires three conditions: (1) glutamate binding to GluN2, (2) glycine/D-serine binding to GluN1, and (3) postsynaptic depolarization to relieve voltage-dependent Mg^2+^ block of the channel pore. Mechanistically, Mg^2+^ blocks the NMDAR pore by binding near a narrow selectivity filter called the “Asn-cage”, which is formed by conserved asparagine residues in the GluN1 and GluN2 subunits, including GluN1a Asn616 and GluN2B Asn615. Mg^2+^ remains fully hydrated and cannot pass through this filter. Instead, it creates a water-mediated interaction network that physically occludes the pore. In contrast, Ca^2+^ can permeate the channel because it undergoes partial dehydration as it passes through multiple binding sites within the Asn-cage (Steigerwald et al., 2026).

The difference between Mg^2+^ block and Ca^2+^ permeation is reflected by the higher dehydration energy of Mg^2+^ compared with Ca^2+^. In addition, tightly bound phospholipids located near the selectivity filter can further influence the voltage sensitivity of Mg^2+^ block (Steigerwald et al., 2026). Once activated, NMDARs conduct calcium (Ca^2+^), sodium (Na^+^), and potassium (K^+^) ions, with calcium influx serving as the primary trigger for downstream signaling (Traynelis et al., 2010).

The amplitude and dynamics of Ca^2+^ signals dictate the direction of plasticity, with larger increases typically promoting long-term potentiation (LTP; high-frequency stimulation, e.g., 100 Hz tetanus) and smaller or prolonged signals favoring long-term depression (LTD; low-frequency stimulation, e.g., 1 Hz for 15 min) (Evans and Blackwell, 2015). In addition, Ca^2+^-dependent activation of kinases such as CaMKII, protein kinase A (PKA), protein kinase C (PKC), and Src regulates NMDAR trafficking, increasing or decreasing their synaptic expression (NMDAR-LTP and NMDAR-LTD) (Traynelis et al., 2010; Lisman et al., 2012). Thus, CaMKII phosphorylates AMPA receptors (GluA1 subunit at Ser831), increasing single-channel conductance and promoting AMPAR insertion into the postsynaptic membrane (Mammen et al., 1997; Kristensen et al., 2011). CaMKII also phosphorylates synaptic scaffolding proteins (PSD-95, SAP102), stabilizing enlarged spines (Tsui and Malenka, 2006; Dosemeci and Jaffe, 2010; Yamada et al., 2019). In turn, Ca^2+^-dependent activation of adenylyl cyclase, PKA, and CREB drives transcription of plasticity genes (BDNF, Arc, Homer1a), consolidating LTP (Benito and Barco, 2010). Beyond ion flux, NMDARs exert non-canonical signaling functions, in which ligand binding alone can trigger intracellular pathways that lead to LTD and structural changes, such as dendritic spine shrinkage. LTD mediated by extrasynaptic GluN2B-containing NMDARs may couple to DAPK1 and trigger spine shrinkage (Stein et al., 2020; Dupuis et al., 2023; Barnes et al., 2025). Furthermore, the nanoscale organization and dynamic redistribution of NMDARs within synapses, as well as their interactions with signaling proteins such as CaMKII, shape synaptic strength, metaplasticity, and ultimately learning and memory.

#### 3.1.2 Synaptic vs. extrasynaptic NMDA composition

A critical yet often underappreciated distinction in glutamatergic neurotransmission exists between synaptic NMDARs and extrasynaptic NMDARs (located perisynaptically or on dendritic shafts) (Hardingham and Bading, 2010; Paoletti et al., 2013), which differ in their subunit composition, activation patterns, downstream signaling cascades, and functional consequences. Understanding this spatial dichotomy is essential for explaining the pathophysiology of depression and the mechanism of action of rapid-acting antidepressants such as ketamine. The concept of synaptic vs. extrasynaptic NMDAR signaling proposes that synaptic NMDAR activation promotes cell survival, whereas extrasynaptic NMDAR activation promotes cell death (Hardingham and Bading, 2010). This “location-dependent signaling” paradigm has since been extended to depression, where chronic stress and inflammation preferentially activate extrasynaptic receptors, triggering pro-apoptotic and anti-plasticity cascades (Lau and Zukin, 2007; Duman and Aghajanian, 2012).

Synaptic NMDARs, enriched in GluN2A subunits, are anchored within the postsynaptic density by scaffolding proteins including PSD-95 and SAP102 (Sans et al., 2000); they are activated by phasic glutamate release from presynaptic terminals during normal neurotransmission, and their activation is tightly regulated by both temporal and spatial constraints (Traynelis et al., 2010). In turn, extrasynaptic NMDARs, which are preferentially enriched in GluN2B subunits, are located perisynaptically, on dendritic shafts, and on the cell soma (Sanz-Clemente et al., 2013). Extrasynaptic NMDARs are activated by tonic glutamate during pathological conditions such as chronic stress and neuroinflammation, when astrocytic glutamate clearance is impaired, and extracellular glutamate levels rise.

The spatial segregation of NMDAR signaling provides an elegant mechanistic framework for understanding how chronic stress and neuroinflammation produce the structural and functional brain changes observed in depression (Duman and Aghajanian, 2012).

In depression, NMDAR function is dysregulated in a region-specific manner. Numerous studies report heterogeneous alterations in the composition of GluN2 subunits that vary across brain regions and experimental models. Stress and depression also modify NMDAR localization, such as by increasing synaptic GluN2B-containing receptors, thereby influencing synaptic plasticity and glutamatergic signaling. These NMDAR alterations are associated with changes in intracellular pathways, including mTOR and GSK-3β, which regulate synaptic protein synthesis and the balance between excitation and inhibition in the brain (Karolewicz et al., 2005; Feyissa et al., 2009; Karolewicz et al., 2009; Chandley et al., 2014; Treccani et al., 2016; Xia et al., 2016; Bieler et al., 2021; Elhussiny et al., 2021; Zhang et al., 2021; Vestring et al., 2025).

#### 3.1.3 Mechanism

##### 3.1.3.1 Molecular mechanism of NMDAR blockade

Ketamine, as an uncompetitive NMDAR antagonist, binds within the ion channel pore of the cell membrane exclusively when the channel is open. This use-dependent blockade confers selectivity for tonically active NMDARs over phasically active synaptic NMDARs (Krystal et al., 2024). Cryo-electron microscopy studies have demonstrated that ketamine binds to the transmembrane domain (TMD) of this protein complex, a hydrophobic pocket within the NMDAR channel, formed by residues Leu642 of GluN2A, Leu643 of GluN2B, and Asn616 of GluN1 (Zhang et al., 2021). This interaction physically occludes ion permeation and stabilizes the closed-channel conformation.

##### 3.1.3.2 Disinhibition hypothesis and interneuron selectivity

A central tenet of ketamine's mechanism is its preferential antagonism of NMDARs located on GABAergic interneurons, particularly somatostatin (SST)- and parvalbumin (PV)-expressing interneurons in the PFC and hippocampus (Gerhard et al., 2020; Homayoun and Moghaddam, 2007). This selectivity is thought to arise from several factors, including higher tonic NMDAR activity in interneurons compared with pyramidal neurons, driven by sustained glutamatergic input and a higher NMDAR/AMPAR ratio (Krystal et al., 2024). As a result, interneuronal NMDARs are more susceptible to use-dependent blockade by ketamine. Inhibition of these interneurons reduces GABAergic tone onto pyramidal neurons, leading to disinhibition and increased cortical excitability (Homayoun and Moghaddam, 2007) (see Figure 3). PV interneurons, in particular, are highly metabolically active fast-spiking cells, which may further increase their vulnerability to NMDAR blockade (Krystal et al., 2024). Additionally, interneurons express higher levels of GluN2B-containing NMDARs, which show greater ketamine sensitivity, and may lack robust homeostatic mechanisms to compensate for receptor inhibition (Gerhard et al., 2020).

**Figure 3:**
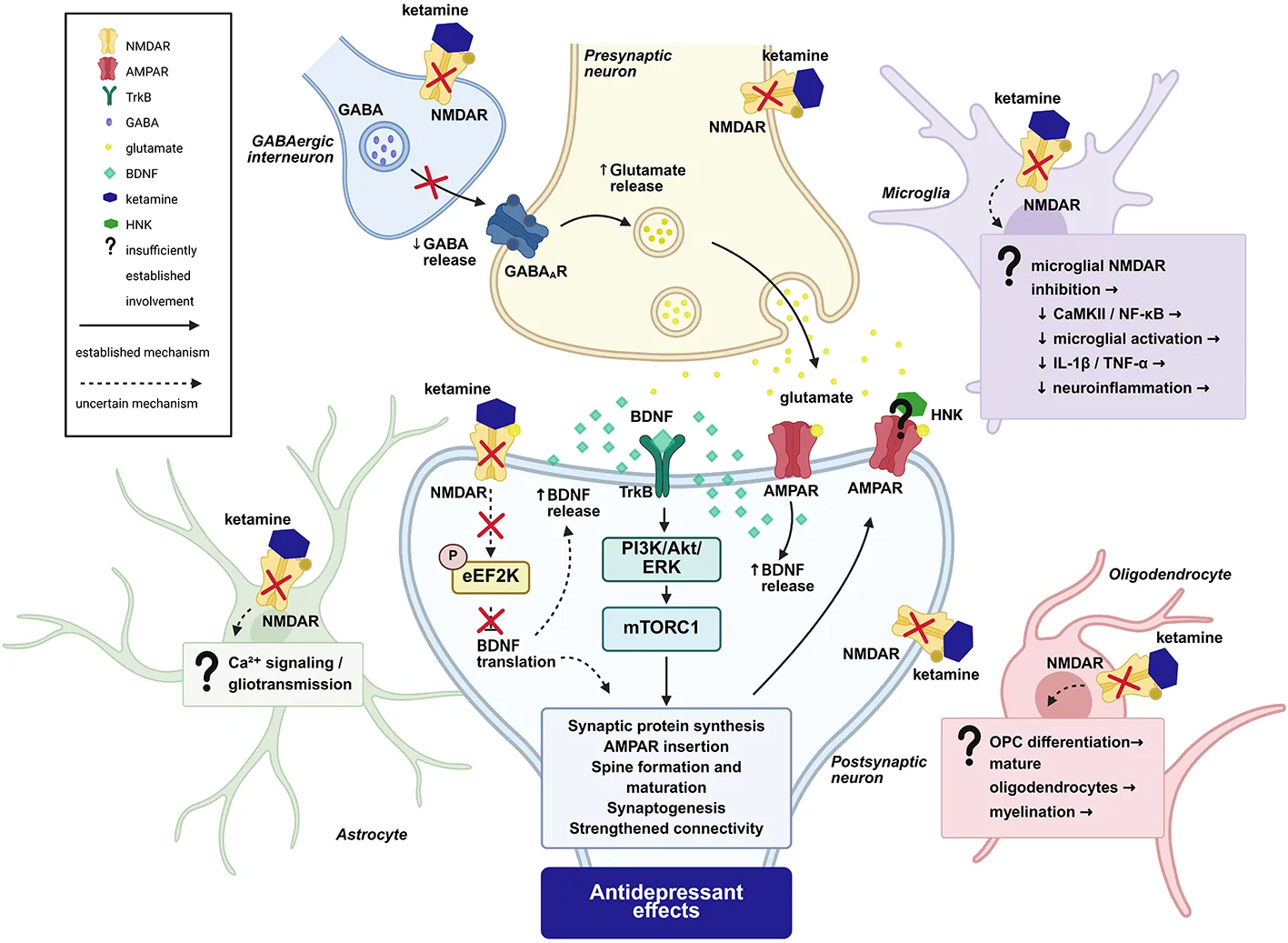
The proposed cellular and molecular mechanisms underlying ketamine's antidepressant effects involve NMDAR-dependent signaling in neurons and glial cells. In GABAergic neurons, ketamine-mediated NMDAR blockade may reduce GABA release. This leads to disinhibition of excitatory neurons and increased presynaptic glutamate release. Enhanced glutamatergic transmission promotes postsynaptic AMPAR activation and BDNF release. This is followed by TrkB-dependent activation of PI3K/Akt/ERK and mTORC1 signaling pathways. Concurrently, blockade of postsynaptic NMDARs may suppress eEF2K activity and facilitate BDNF translation. These convergent pathways promote synaptic protein synthesis, AMPAR membrane insertion, dendritic spine formation and maturation, synaptogenesis, and strengthening of synaptic connectivity. Together, they contribute to the rapid and sustained antidepressant effects of ketamine. Ketamine metabolites, such as hydroxynorketamine (HNK), may further facilitate AMPAR-dependent signaling, although the mechanisms remain incompletely understood. The potential contributions of glial NMDARs are less well characterized. In astrocytes, NMDAR modulation may influence intracellular Ca^2+^ signaling and gliotransmission. In microglia, NMDAR inhibition has been proposed to attenuate CaMKII/NF-κB signaling, microglial activation, and pro-inflammatory cytokine production. In oligodendrocyte-lineage cells, NMDAR modulation may affect OPC differentiation, oligodendrocyte maturation, and myelination. Solid arrows represent well-established mechanisms, while dashed arrows and question marks indicate proposed or insufficiently established pathways. Created in BioRender. Sulej, Z. (2026).

Clinical and neuroimaging studies support the functional relevance of this mechanism, as MRS findings indicate that changes in neurotransmitter levels in the ACC are associated with better antidepressant response to ketamine (Milak et al., 2020). Conversely, elevated inflammatory markers (e.g., CRP ≥ 3 mg/L) have been linked to reduced treatment response or the need for adjunctive anti-inflammatory strategies (Chen et al., 2025).

##### 3.1.3.3 AMPAR activation and the role of hydroxynorketamine metabolites

Disinhibition leads to increased firing rates of pyramidal neurons and induces a surge of glutamate release onto neighboring pyramidal neurons, thereby activating postsynaptic AMPARs (Krystal et al., 2024) (see Figure 3). This glutamate surge is essential for ketamine's antidepressant effects, as AMPAR antagonists inhibit ketamine-induced synaptogenesis and behavioral responses (Li et al., 2010). Pharmacological blockade of AMPARs with antagonists such as NBQX and CNQX completely abolishes ketamine-induced mTORC1 activation, BDNF release, and dendritic spine formation, highlighting the indispensable role of AMPARs in downstream signaling and structural plasticity (Li et al., 2010; Aleksandrova et al., 2017).

The significance of AMPAR signaling has led to the hypothesis that AMPAR potentiation alone may be sufficient to produce rapid antidepressant effects, independent of NMDAR blockade. Supporting this hypothesis, AMPAR positive allosteric modulators (AMPAkines) induce rapid antidepressant-like effects in preclinical models (Aleksandrova et al., 2017). However, clinical trials of AMPAkines have produced mixed outcomes, potentially due to issues with brain penetration, suboptimal dosing, or the complexity of translating preclinical findings to human depression (Yang et al., 2019). Nonetheless, agents that facilitate AMPAR activation may enhance ketamine's efficacy (Aleksandrova et al., 2017), positioning AMPARs as promising targets for next-generation rapid-acting antidepressants. Additional evidence for the importance of AMPAR signaling is provided by studies of ketamine's active metabolite, (2R,6R)-hydroxynorketamine (HNK). Ketamine is rapidly metabolized by hepatic cytochrome P450 enzymes, and HNK attains brain concentrations in the low micromolar range sufficient to modulate synaptic function. In a landmark 2016 study, Gould, Zarate, and colleagues proposed that HNK, rather than ketamine itself, mediates the antidepressant response. HNK elicits robust antidepressant-like effects in rodent models (e.g., forced swim test, learned helplessness, chronic social defeat stress) and enhances AMPAR-mediated synaptic transmission and LTP in the hippocampus (Zanos et al., 2016). Furthermore, HNK increases dendritic spine density and mTORC1 signaling in the PFC (Li et al., 2010; Paul et al., 2014; Yang C. et al., 2018).

Notably, HNK appears to lack the adverse effects associated with ketamine. It does not induce dissociative or psychotomimetic effects, nor does it cause locomotor hyperactivity, stereotypy, or conditioned place preference, indicating a low potential for abuse (Zanos et al., 2016). These findings suggest that HNK exerts its therapeutic actions through AMPAR-dependent mechanisms while functioning independently of NMDAR antagonism, thereby offering a potential pathway to antidepressant efficacy without the side effects linked to NMDAR blockade (Zhou et al., 2013; Fukumoto et al., 2019).

Several independent research groups have failed to replicate the claim that HNK lacks NMDAR antagonist activity (Zhang et al., 2018; Zanos et al., 2023). At brain-relevant concentrations (1–10 μM), HNK has been shown to block NMDARs, although with lower potency than ketamine. In addition, some studies indicate that its antidepressant-like effects can be inhibited by NMDAR agonists or require basal NMDAR activity, suggesting that these effects are not entirely NMDAR-independent (Zanos et al., 2023). Genetic deletion of the GluN2D subunit also abolishes sustained antidepressant-like effects, further supporting a role for NMDAR function (Yamagishi et al., 2024).

The pharmacokinetics and translational relevance of HNK in humans also remain unclear, as its brain penetration and synaptic concentrations are still unknown despite its detectability in plasma after ketamine infusion (Zhang et al., 2018). Overall, the mechanism and clinical significance of HNK remain under active investigation and debate (Jiang et al., 2024).

##### 3.1.3.4 Intracellular signaling: eEF2/BDNF/mTORC1 cascade

In addition to the interneuron-disinhibition model, substantial preclinical evidence supports a direct contribution of NMDARs expressed by excitatory pyramidal neurons to ketamine's effects (Krystal et al., 2024; Park et al., 2026). This mechanism has been particularly well characterized in the hippocampus, where suppression of spontaneous NMDAR-mediated neurotransmission in the CA1 region induces rapid, protein synthesis-dependent synaptic potentiation (Autry et al., 2011; Nosyreva et al., 2013).

Studies involving cortical principal neurons further support a role of excitatory-neuron NMDARs in ketamine action. Deletion of GluN2B from cortical principal neurons mimicked and occluded several ketamine actions, including enhanced excitatory synaptic transmission, mTOR activation, and increased synaptic protein synthesis. These findings suggest that basal signaling through GluN2B-containing NMDARs contributes to the regulation of excitatory synaptic strength in the PFC (Miller et al., 2014).

Recent research has expanded this model by implicating GluN2A-containing NMDARs. Su et al. (2023) showed that deletion of GluN2A in excitatory, but not inhibitory, neurons produced antidepressant-like behavioral responses. Additionally, hippocampal GluN2A knockdown was sufficient to induce antidepressant-like effects, while acute ketamine or MK-801 treatment rapidly increased the intrinsic excitability of CA1 pyramidal neurons in a GluN2A-dependent manner. These results indicate that NMDARs located directly on excitatory neurons can contribute to ketamine-induced behavioral and cellular responses and suggest that different NMDAR subpopulations may participate depending on the neuronal population and experimental context (Su et al., 2023).

At the intracellular level, these receptor-mediated effects converge on signaling pathways that regulate rapid protein translation and synaptic plasticity. Under basal conditions, spontaneous NMDAR-mediated neurotransmission maintains eEF2K-dependent phosphorylation of the translation elongation factor eEF2. Ketamine-mediated suppression of this signaling reduces eEF2 phosphorylation, thereby de-repressing BDNF translation from dendritic mRNA stores, leading to increased BDNF availability and subsequent TrkB-dependent signaling (Adaikkan et al., 2018; Autry et al., 2011).

BDNF acts in an autocrine or paracrine manner to activate TrkB receptors on the same or neighboring neurons, initiating intracellular signaling cascades that enhance synaptic strength and promote AMPAR insertion into the postsynaptic membrane (Kowiański et al., 2018; Colucci-D'Amato et al., 2020). BDNF binding induces TrkB receptor dimerization and autophosphorylation of intracellular tyrosine residues. Phosphorylated TrkB recruits adaptor proteins (Shc, Grb2) and activates multiple downstream pathways (Numakawa et al., 2018). TrkB activation stimulates phosphoinositide 3-kinase (PI3K), which phosphorylates phosphatidylinositol (4,5)-bisphosphate (PIP_2_) to generate phosphatidylinositol (3,4,5)-trisphosphate (PIP_3_). PIP_3_ recruits protein kinase B (Akt) to the membrane, where it is phosphorylated and activated by PDK1 and mTORC2. Increased glutamatergic activity and BDNF-TrkB signaling further stimulate key plasticity-related pathways, including the mammalian target of rapamycin complex 1 (mTORC1), a central regulator of protein synthesis, cell growth, and synaptic plasticity, as well as kinases ERK and Akt (Ignácio et al., 2016; Levy et al., 2018). These pathways collectively promote synaptogenesis, dendritic spine formation, and strengthen synaptic connectivity in mood-relevant brain regions, particularly the PFC and hippocampus (Yang et al., 2020). Akt phosphorylates and inhibits tuberous sclerosis complex 2 (TSC2), relieving inhibition of Rheb (Ras homolog enriched in brain), a small GTPase that directly activates mTORC1 (Li et al., 2010).

Importantly, recent evidence has fundamentally expanded this understanding by demonstrating that ketamine and its metabolites directly bind to TrkB neurotrophin receptors, positioning TrkB not merely as a downstream effector but as a proposed molecular target (Duman, 2018; Krystal et al., 2024). Using atomistic molecular dynamics simulations, Casarotto and colleagues identified a binding site located at the TMD of TrkB dimers. Ketamine directly binds to this site with micromolar affinity (*Ki* = 12.30 μM), a value that falls within the range of brain ketamine concentrations achieved following an antidepressant dose (~6–11 μM), supporting the physiological relevance of this interaction (Casarotto et al., 2021).

Mechanistically, ketamine acts as a molecular “wedge” that stabilizes the cross-shaped active conformation of TrkB TMD dimers. The TrkB transmembrane domain possesses a cholesterol-sensing function that mediates synaptic effects of cholesterol on receptor conformation; ketamine binding at this site is particularly effective under conditions of high cholesterol content, such as those found in synaptic membranes (Casarotto et al., 2021). By stabilizing the active dimeric conformation, ketamine promotes TrkB translocation to the cell surface and its localization at synaptic sites, where it becomes accessible to BDNF for activation.

The potential functional significance of this interaction was further supported by studies using a point mutation (Y433F) in the proposed TrkB antidepressant-binding motif. In wild-type mice, ketamine administration yields robust memory enhancement, rapid antidepressant-like responses, and promotion of contextual fear extinction. All of these behavioral benefits are lost in TrkB.Y433F mutant mice. Furthermore, ketamine rapidly reactivates critical period-like plasticity in the adult visual cortex, inducing ocular dominance shifts following monocular deprivation, but this plasticity was abolished in TrkB.Y433F mutant mice, supporting an important role for this TrkB motif in the observed response (Casarotto et al., 2021).

At the cellular level, ketamine promotes the formation of TrkB clusters at the plasma membrane, enhances receptor phosphorylation at the Y816 residue in primary cortical neurons, and drives TrkB trafficking directly into dendritic spines in hippocampal neurons. This signaling cascade upregulates the surface expression of AMPA receptors, a critical requirement for synaptic potentiation (Aleksandrova et al., 2017). In TrkB.Y433F mutant model, ketamine fails to bind TrkB, and its ability to promote dendritic spine trafficking and AMPAR membrane insertion is completely abolished (Casarotto et al., 2021).

Both esketamine and the HNK can also bind TrkB and facilitate its activation by BDNF, suggesting that TrkB engagement extends to metabolites implicated in the sustained antidepressant response (Casarotto et al., 2021; Moliner et al., 2023).

mTORC1 activation, whether triggered through the indirect eEF2-mediated BDNF release or through direct TrkB binding, drives rapid translation of synaptic proteins, including GluA1 subunit, PSD-95 (a postsynaptic scaffolding protein), Arc (an activity-regulated cytoskeleton-associated protein), and synapsin (a presynaptic vesicle protein) (Ignácio et al., 2016; Li et al., 2010). Activated mTORC1 phosphorylates p70S6 kinase and eIF4E-binding protein 1 (4E-BP1), both essential for translation of these synaptic proteins (Li et al., 2010). Newly synthesized proteins are transported to dendritic spines, where, within 24 h, a significant increase in the density of mushroom-shaped dendritic spines is observed in layer V pyramidal neurons of the PFC, correlating with antidepressant behavioral responses (Li et al., 2010). Pharmacological inhibition of mTORC1 with rapamycin prevents ketamine-induced synaptogenesis and antidepressant effects, confirming the necessity of this pathway (Li et al., 2010).

Clinical findings add further nuance; in ketamine treated patients with PTSD at mid-treatment, 1-week follow-up, and 1-month follow-up, small but significant decreases in BDNF levels were detected (Quigley et al., 2026). This suggests that initial decreases in BDNF levels during ketamine treatment may reflect normalization of neurotrophic signaling as symptoms improve, rather than treatment failure, consistent with the understanding that BDNF increases in reaction to stress-induced neuronal damage but decreases when the stressor is mitigated (Duncan et al., 2013; Haile et al., 2014). A significant positive correlation between BDNF and VEGF-A has been observed at all time points during ketamine treatment, and rodent studies demonstrate that sustained antidepressant action requires reciprocal interdependence of both neurotrophic factors (Quigley et al., 2026).

Finally, Casarotto et al. (2021) proposed that binding to TrkB and allosteric facilitation of BDNF signaling represent a common mechanism for antidepressant action shared by both conventional and fast-acting agents. This framework may explain why typical antidepressants act slowly, requiring weeks of treatment to achieve sufficient TrkB occupancy and downstream plasticity, while ketamine, with its higher binding efficacy and concurrent NMDAR-mediated glutamate burst, produces rapid effects (Browne and Lucki, 2013; Gould et al., 2019).

In addition to these glutamatergic and neurotrophic mechanisms, emerging evidence suggests ketamine's antidepressant effects may also involve indirect engagement of adenosine A1 and A2A receptors (A1R/A2AR) (Yue et al., 2026; Dautan et al., 2026). Ketamine rapidly alters neuronal and astrocytic energy metabolism in the mPFC, lowering intracellular ATP/ADP ratios and driving nucleoside transporters ENT1/2-mediated adenosine efflux, independent of NMDAR activity. The resulting extracellular adenosine surge engages A1R/A2AR signaling that appears necessary for ketamine's antidepressant-like effects and downstream BDNF induction in preclinical models (Serchov et al., 2015; Lazarevic et al., 2021; Yue et al., 2026; Dautan et al., 2026). This metabolic–purinergic pathway may act in parallel with, rather than instead of, the canonical NMDAR–AMPAR–BDNF signaling cascade, representing a promising direction for future mechanistic work.

##### 3.1.3.5 Plasticity and synaptogenesis

Newly synthesized synaptic proteins are transported to dendritic spines, where they stabilize and enlarge the postsynaptic density. Within 2–4 h of ketamine administration, a significant increase in the density of mushroom-shaped dendritic spines (mature, stable spines with large heads and narrow necks) is observed in layer V pyramidal neurons of the PFC, correlating with antidepressant behavioral responses (Li et al., 2010).

Pharmacological inhibition of mTORC1 with rapamycin prevents ketamine-induced synaptogenesis and antidepressant effects, confirming the necessity of this pathway (Li et al., 2010; Yang et al., 2019; Abdallah et al., 2020). Similarly, BDNF-TrkB signaling is essential, as TrkB antagonists or BDNF knockdown block ketamine's effects (Yang et al., 2019).

### 3.2 Beyond neurons: NMDA in glia

The traditional neuron-centric view of depression has been challenged by compelling evidence implicating glial cells: astrocytes, microglia, and oligodendrocytes as critical players in its pathophysiology (Rajkowska and Miguel-Hidalgo, 2007; Sanacora and Banasr, 2013). Glial dysfunction contributes to disrupted synaptic homeostasis, impaired neuroplasticity, neuroinflammation, and white matter abnormalities, collectively underpinning the core features of e.g., MDD.

Astrocytes, the most abundant glial cell type in the brain, play indispensable roles in glutamate clearance, energy metabolism, blood-brain barrier maintenance, and synaptic modulation (Allen and Barres, 2009; Czéh and Nagy, 2018; Verkhratsky et al., 2019). Postmortem studies consistently demonstrate reduced astrocytic density and volume in key mood-regulating regions of depressed patients, including the PFC, the ACC, and hippocampus (Rajkowska et al., 1999; Miguel-Hidalgo et al., 2000; Cotter et al., 2001). Neuroimaging studies corroborate these findings, revealing decreased glial cell numbers and altered astrocytic morphology *in vivo* (Bowley et al., 2002). Additionally, postmortem and neuroimaging studies reveal reduced expression of astrocytic glutamate transporters, particularly GLT-1/EAAT2, in the PFC and hippocampus of depressed patients (Choudary et al., 2005; Bernstein et al., 2015). Decreased glutamine synthetase (GS) activity further disrupts the glutamate-glutamine cycle, leading to extracellular glutamate accumulation and excitotoxicity (Banasr and Duman, 2008; Son et al., 2018; Baek et al., 2019). Observed deficits contribute to the elevated glutamate levels observed via MRS in depressed patients (Luykx et al., 2012), as well as to the loss of synapse-related gene expression and synaptic density in the PFC (Kang et al., 2012).

Beyond astrocytes that fail to maintain synaptic homeostasis, microglia that orchestrate neuroinflammation are recognized as key mediators of the inflammatory hypothesis of depression (Yirmiya et al., 2015), undergoing a transition from a homeostatic to a pro-inflammatory, activated state, releasing cytokines and contributing to depressive symptoms (Köhler et al., 2014; Miller and Raison, 2016).

Also, there is substantial evidence suggesting impairment of oligodendrocytes and oligodendrocyte progenitor cells (OPCs) responsible for myelin formation and maintenance (Bergles and Richardson, 2016). The evidence comes from animal models of chronic stress, in which decreases in these cells have been noted in the PFC, and several studies in human and preclinical models have noted white matter and myelination abnormalities (Edgar and Sibille, 2012). In postmortem human tissue of patients with MDD, single-cell transcriptomic analysis revealed that almost half of the gene expression changes in the medial prefrontal cortex (mPFC) were related to OPCs (Nagy et al., 2020).

While the global dysfunction of glial cells is now increasingly recognized as a fundamental contributor to the pathophysiology of depression, specific molecular mechanisms driving these impairments, engaging signaling machinery, specifically NMDA receptors, may be key targets for understanding how synaptic homeostasis is disrupted and subsequently restored.

#### 3.2.1 NMDA receptors in astrocytes

Astrocytes express functional NMDARs (Lalo et al., 2006; Lee et al., 2010; Palygin et al., 2011; Montes De Oca Balderas and Aguilera, 2015; Verkhratsky et al., 2019; Verkhratsky and Chvátal, 2020). Analysis of gene expression in cells from mouse and human primary cultures demonstrated that astroglial cells express all NMDA receptor subunits (GluN1, GluN2A, GluN2B, GluN2C, GluN2D, GluN3A, and GluN3B), with GluN1, GluN2C, and GluN3A being the most abundant (Cahoy et al., 2008; Lee et al., 2010; Montes De Oca Balderas and Aguilera, 2015; Verkhratsky et al., 2019; Cuellar-Santoyo et al., 2023). The occurrence of NMDARs in astroglial cells has been confirmed by multiple immunohistochemical investigations. Protein expression of the obligatory GluN1 subunit, along with several GluN2 variants, has been detected in cultured astrocytes (Zhou et al., 2010; Gérard and Hansson, 2012; Skowrońska et al., 2019b). In the basolateral amygdala, electron microscopy studies using antibodies against GluN1 indicate that roughly one-fifth of the labeled structures correspond to distal astrocyte processes (Gracy and Pickel, 1995). Comparable findings have been reported in the adult rat cortex, where both distal processes and, occasionally, the cell bodies of astrocytes show immunoreactivity for GluN1 and GluN2A/B subunits (Conti et al., 1996; Verkhratsky et al., 2019).

Additionally, astroglial processes located near synapses, marked by GluN1 and GluN2A/B, have been identified in the dorsal cochlear nucleus (Petralia et al., 1996; Verkhratsky et al., 2019). GluN1-positive astrocytic processes have also been observed in regions such as the locus coeruleus and the basal nuclei of the amygdala (Farb et al., 1995; Van Bockstaele and Colago, 1996; Dzamba et al., 2013). Furthermore, studies employing a fluorescent tagging approach, in which EGFP is fused to the GluN2C subunit, have demonstrated astroglial expression of GluN2C in brain areas including the cortex, striatum, hippocampus, and amygdala, whereas no such expression was observed in the thalamus or basal ganglia (Ravikrishnan et al., 2018).

In astrocytes, NMDARs function differently from those in neurons, activated by glutamate and operate via two distinct mechanisms: ionotropic and metabotropic (Lalo et al., 2006; Palygin et al., 2011; Brunetti et al., 2024). Ionotropically, channel opening allows Ca^2+^ influx, whereas metabotropically, no ion flow occurs; instead, intracellular signaling pathways involving phospholipase C (PLC), inositol trisphosphate (IP_3_), and ryanodine receptor-mediated pathways trigger Ca^2+^ release from the endoplasmic reticulum (ER) (Gérard and Hansson, 2012; Montes De Oca Balderas and Aguilera, 2015). Astrocytic NMDAR-mediated currents are smaller than neuronal ones and appear to be largely Mg^2+^-independent (Lalo et al., 2006; Palygin et al., 2011; Kosenkov et al., 2024). Most authors attribute this property to the incorporation of the GluN3 subunit, as receptors containing GluN1/GluN3 have been shown to exhibit reduced Mg^2+^ sensitivity (Dzamba et al., 2013; Paoletti et al., 2013). However, to date, there is no definitive structural or protein-level evidence demonstrating fully functional GluN3-containing NMDARs in astrocytes. Therefore, it remains possible that the reduced Mg^2+^ block is instead due to the presence of GluN2C/D subunits, which exhibit relatively weak voltage-dependent Mg^2+^ inhibition and can therefore conduct current at resting membrane potentials (Palygin et al., 2011; Paoletti et al., 2013).

Activation of astrocytic NMDARs may trigger intracellular Ca^2+^ elevations that can propagate within individual astrocytes and, under certain conditions, across astrocytic networks as Ca^2+^ waves. This propagation coordinates responses over broader spatial domains (Bazargani and Attwell, 2016; Goenaga et al., 2023). These Ca^2+^ signals regulate diverse astrocytic functions, such as gliotransmitter release, metabolic support, and modulation of local vascular responses (Bazargani and Attwell, 2016; Mahmoud et al., 2019; Goenaga et al., 2023). Specifically, Ca^2+^-dependent activation of vesicular release machinery facilitates SNARE-dependent exocytotic release of glutamate, ATP, and D-serine (Bezzi et al., 2004; Martineau et al., 2013; Lalo et al., 2014), influencing adjacent neuronal networks.

Astrocyte-derived glutamate can modulate neuronal NMDARs and AMPARs, while D-serine regulates the availability of the NMDAR co-agonist and contributes to NMDAR-dependent synaptic plasticity (Pirttimaki et al., 2011; Robin et al., 2018; Koh et al., 2022). ATP released from astrocytes also serves as a functionally relevant downstream signal. Supporting this, selective reduction of NMDAR expression in cortical astrocytes diminishes sensory-evoked astrocytic Ca^2+^ responses and disrupts nearby neuronal activity, including neuronal recruitment, synchronization, and adaptation. These neuronal and behavioral deficits can be reversed by exogenous ATP, highlighting a role for astrocytic NMDARs in purinergic astrocyte–neuron communication *in vivo* (Ahmadpour et al., 2024).

In addition to acute modulation of synaptic transmission, NMDAR-dependent Ca^2+^ signaling may influence long-term astrocytic homeostatic functions. Prolonged NMDA exposure reduces the expression of GS, AQP4, and Kir4.1 in cortical astrocytes in a Ca^2+^- and GluN1-dependent manner, potentially affecting glutamate–glutamine metabolism, water homeostasis, and extracellular potassium buffering (Skowrońska et al., 2019a). NMDAR activation is further associated with alterations in mitochondrial membrane potential, induction of endoplasmic reticulum stress, and increased astrocytic glutathione synthesis (Jimenez-Blasco et al., 2015; Montes De Oca Balderas and Aguilera, 2015; Montes De Oca Balderas et al., 2020).

These findings indicate that astrocytic NMDAR signaling extends beyond gliotransmission to regulate cellular metabolism and neuroprotective capacity (Jimenez-Blasco et al., 2015; Montes De Oca Balderas and Aguilera, 2015; Skowrońska et al., 2019a).

##### 3.2.1.1 Ketamine mechanism of action via astrocytic NMDAR

Considering the role of astrocytic NMDARs in regulating intracellular Ca^2+^ signaling, astrocyte–neuron communication, and extracellular homeostasis modulation may be part of ketamine's mechanism of action. Ketamine influences astrocytic function through several partially independent pathways that may converge on regulating neuronal excitability, synaptic plasticity, and extracellular homeostasis (Figure 3). These effects include changes in membrane organization and cAMP signaling; modulation of astrocytic Ca^2+^ dynamics and vesicle–plasmalemma interactions; regulation of Kir4.1-dependent K^+^ buffering and GLT-1-mediated glutamate clearance; and changes in astrocytic metabolic support (Wang, 2019; Ouyang et al., 2021). However, current evidence shows that most of these effects have not been directly linked to the blockade of astrocytic NMDARs, and some involve NMDAR-independent mechanisms.

Nevertheless, astrocytic NMDARs may still be relevant to specific ketamine-induced astrocytic responses. However, the extent to which direct blockade of astrocytic NMDARs contributes to these effects, and to ketamine's rapid antidepressant actions, remains undetermined.

##### 3.2.1.2 Ketamine NMDAR-independent mechanisms

###### 3.2.1.2.1 Cholesterol, cAMP-PKA-CREB signaling and plasticity gene expression

Ketamine, an amphipathic molecule, interacts with the astrocytic plasma membrane and induces the redistribution of cholesterol-rich membrane domains in cultured rat astrocytes following short-term ketamine exposure (Lasič et al., 2019). This reorganization may promote the relocation of Gαs from cholesterol-rich lipid rafts to non-raft regions, enhancing its interaction with adenylyl cyclase and increasing intracellular cAMP. Elevated cAMP activates PKA, which phosphorylates CREB at Ser133 and regulates the expression of BDNF and other synaptic plasticity-related genes (Wray et al., 2019). Supporting this mechanism, lipid-raft fractionation and fluorescence recovery after photobleaching analyses in C6 glioma cells showed that ketamine rapidly redistributed Gαs from lipid rafts. Functional assays with a cAMP biosensor confirmed increased Gαs-dependent cAMP signaling. This effect was not seen with other NMDAR antagonists (MK-801, memantine, AP-V) and persisted after NR1 knockdown, indicating an NMDAR-independent mechanism. In primary rat astrocytes, ketamine exposure increased BDNF levels 24 h after exposure, whereas pharmacological inhibition of cAMP signaling reduced this effect, supporting a cAMP-dependent mechanism for astrocytic BDNF production (Wray et al., 2019). The ketamine metabolite (2R,6R)-hydroxynorketamine also induces Gαs redistribution and increases cAMP levels (Wray et al., 2019), a process that appears to be central to the drug's therapeutic profile. Astrocyte-derived BDNF may contribute to extracellular BDNF availability and TrkB-dependent signaling in neighboring neurons, although the extent of this contribution *in vivo* remains to be established (Stenovec, 2021).

BDNF may also regulate astrocytic cholesterol homeostasis. In human astrocyte models, BDNF increased cholesterol efflux and upregulated ABCA1 and ApoE, both of which are involved in cholesterol export (Spagnuolo et al., 2018). Independently, glia-derived cholesterol transported in ApoE-containing lipoproteins has been shown to promote synapse formation and neuronal maturation (Mauch et al., 2001). These findings suggest that BDNF-dependent regulation of astrocytic cholesterol efflux may increase the availability of glia-derived cholesterol, thereby contributing to membrane supply and providing trophic support for synaptogenesis and structural plasticity. However, a direct BDNF-dependent increase in astrocyte-to-neuron cholesterol transfer has not yet been demonstrated (Mauch et al., 2001; Suzuki et al., 2007; Stenovec et al., 2020; Stenovec, 2021).

Modulation of these astrocytic functions may stabilize the extracellular environment, support the prolonged effects of ketamine on neuronal circuits, and contribute to its sustained antidepressant efficacy.

###### 3.2.1.2.2 Kir4.1-mediated mechanism in the lateral habenula

Ketamine-induced cAMP signaling may regulate other astrocytic homeostatic processes, including Kir4.1 trafficking, thereby providing a potential link between ketamine-induced astrocytic signaling and extracellular K^+^ homeostasis (Božić et al., 2023). In cultured cortical astrocytes, short-term ketamine exposure reduced the mobility of Kir4.1-containing vesicles. This effect was reproduced by dbcAMP and by increased extracellular K^+^, indicating that cAMP signaling is involved (Božić et al., 2023). Furthermore, ketamine reduced the surface density of Kir4.1 and diminished the voltage-activated K^+^ currents, which suggests that decreased vesicular trafficking may limit the availability of Kir4.1 at the astrocyte membrane and thus alter the buffering of extracellular K^+^. This mechanism may be particularly relevant in the lateral habenula (LHb), an epithalamic nucleus that processes aversive stimuli and negative reward-related information. In rodent models of depression, astrocytic Kir4.1 is upregulated in the LHb (Cui et al., 2018). With increased Kir4.1 activity, extracellular potassium clearance is enhanced, decreasing [K^+^]o and causing hyperpolarization of neighboring neurons. This hyperpolarization relieves T-type Ca^2+^ channels from inactivation, so that a subsequent depolarization can trigger T-type Ca^2+^ and NMDAR-dependent currents, resulting in pathological burst firing. Overexpression of Kir4.1 specifically in astrocytes increases bursting in the LHb and leads to depression-like behavior, while a reduction of Kir4.1 suppresses bursting and reduces depression-like behaviors, thereby supporting a causal role for astrocytic K^+^ homeostasis in the excitability of the LHb (Cui et al., 2018).

By reducing Kir4.1 surface availability, ketamine could limit excessive astrocytic K^+^ clearance, thereby opposing the low extracellular K^+^ environment that favors neuronal hyperpolarization and burst firing. At the same time, ketamine acts directly at the neuronal level by blocking NMDAR-dependent bursting in LHb neurons. Both NMDARs and *T*-type Ca^2+^ channels are required for this pathological firing mode, and local NMDAR blockade within the LHb is sufficient to produce rapid antidepressant-like effects (Yang Y. et al., 2018). Thus, modulation of astrocytic Kir4.1-dependent K^+^ homeostasis and direct neuronal NMDAR blockade may represent complementary mechanisms that suppress pathological LHb activity.

The involvement of astrocytic Kir4.1 in the antidepressant effects of ketamine is also supported by pharmacological studies which show that direct inhibition of Kir4.1 can reproduce ketamine-like behavioral responses. The Kir4.1 inhibitor, Lys05, can reverse the depression-like phenotypes caused by Kir4.1 and produces rapid antidepressant-like effects as early as 1 h after administration in various rodent models, with efficacy comparable to that of (S)-ketamine (Zhou et al., 2024). Similarly, Lys05 elicits rapid antidepressant-like responses within 1 h, and these effects disappear upon conditional deletion of Kir4.1 in astrocytes, supporting the dependence of this response on astrocytic Kir4.1 (Wang et al., 2026). These pharmacological results support the notion that suppressing astrocytic Kir4.1 function can mimic certain aspects of ketamine's rapid antidepressant effects and therefore reinforce the hypothesis that ketamine-induced modulation of Kir4.1 trafficking and K^+^ homeostasis may contribute to its rapid antidepressant action.

###### 3.2.1.2.3 Restoration of GLT-1 function and glutamate clearance

GLT-1 (EAAT2) is the major astrocytic glutamate transporter responsible for maintaining low extracellular glutamate levels and limiting glutamate spillover beyond the synaptic cleft. Chronic stress can reduce GLT-1 expression and impair astrocytic glutamate uptake, contributing to excessive extrasynaptic glutamatergic signaling. In a chronic unpredictable stress model in rats, ketamine reversed the stress-induced reduction in GLT-1 expression in the hippocampus, an effect that appeared to involve BDNF–TrkB signaling (Liu et al., 2016).

The functional consequences of GLT-1 downregulation have been shown in the rat medial prefrontal cortex. Chronic stress-induced astrocyte loss and reduced GLT-1 expression impair glutamate uptake and cause extracellular glutamate to overflow onto extrasynaptic GluN2B-containing NMDA receptors. This activates DAPK1, a Ca^2+^/calmodulin-regulated kinase that phosphorylates GluN2B at Ser1303. This triggers excessive calcium influx and inhibits the CREB–BDNF pathway, leading to synaptic protein deficits and depressive-like behavior (Li et al., 2018).

###### 3.2.1.2.4 Calcium signaling and gliotransmitter release

Ketamine can modulate astrocytic calcium signaling through several mechanisms (Stenovec, 2021). Studies in cultured rat astrocytes showed that ketamine markedly attenuates ATP-evoked intracellular Ca^2+^ transients, reducing exocytotic events and BDNF secretion (Stenovec et al., 2016). Ketamine may affect stimulus-evoked Ca^2+^ signaling by modifying Ca^2+^ entry through transient receptor potential (TRP) channels, non-selective cation channels involved in astrocytic Ca^2+^ homeostasis (Stenovec et al., 2020; Stenovec, 2021). Astrocytic NMDAR activation may also induce flux-independent Ca^2+^ release from intracellular stores through IP_3_ receptor-dependent mechanisms, consistent with metabotropic-like NMDAR signaling (Montes De Oca Balderas and Aguilera, 2015). In addition, connexin 43 (Cx43)-mediated gap-junctional coupling and ATP release through connexin hemichannels can contribute to the propagation of intercellular Ca^2+^ signals within astrocytic networks (Stout et al., 2002; Kang et al., 2008).

Astrocytic Ca^2+^ elevations regulate vesicular release of neuroactive signaling molecules, including ATP and, under specific conditions, glutamate and D-serine, through Ca^2+^- and SNARE-dependent mechanisms (Liu Y. et al., 2023). Ketamine also modifies vesicle–plasmalemma interactions via a “flickering fusion pore” mechanism. Using membrane capacitance recordings, Lasič et al. (2016) showed that ketamine increases repetitive fusion-pore opening and closing while reducing irreversible vesicle fission. Ketamine stabilizes the fusion pore in a narrow state, which may restrict efficient cargo discharge and vesicle recycling rather than promote complete exocytosis. Along with reducing stimulus-evoked astrocytic Ca^2+^ signaling, these changes may alter astrocyte–neuron communication and synaptic transmission (Stenovec et al., 2020; Stenovec, 2021).

###### 3.2.1.2.5 Astrocyte-neuron lactate shuttle and metabolic support

The astrocyte-neuron lactate shuttle (ANLS) transports lactate produced by astrocytes to neurons as an energy substrate (Pellerin and Magistretti, 1994). Astrocytes primarily express MCT1 and MCT4, which support lactate export, whereas MCT2 mainly mediates neuronal lactate uptake. Ketamine may influence this coupling by enhancing astrocytic glucose metabolism. In cultured HA1800 human astrocytic cells, ketamine increased glucose uptake and extracellular lactate production (Ouyang et al., 2021). In a chronic unpredictable mild stress model in mice, ketamine also increased glucose uptake in the prefrontal cortex via ERK/GLUT3-dependent signaling (Ouyang et al., 2021). However, whether ketamine-induced astrocytic lactate production directly increases lactate transfer to neurons remains unproven. The potential relevance of this mechanism to depression is supported by evidence that impaired astrocytic glycolysis and reduced lactate production in the prefrontal cortex promote depressive-like behavior. Conversely, astrocyte-derived lactate supports neuronal excitability partly through neuronal MCT2 (Yao et al., 2023). Despite evidence that enhanced astrocytic lactate availability may contribute to the effects of ketamine, a direct causal role of the ANLS in ketamine-induced synaptic plasticity and antidepressant responses has not yet been established.

#### 3.2.2 NMDA receptors in oligodendrocytes

Similar to astrocytes, oligodendrocytes have been reported to express NMDARs (Dzamba et al., 2013). Studies employing immunostaining and Western blot analyses have identified NMDAR subunits, including GluN1, GluN2A, and GluN2B, in A2B5-positive oligodendrocyte precursor cells, O1-positive immature oligodendrocytes, and MBP-positive mature oligodendrocytes in culture (Li et al., 2013). Furthermore, GluN1, GluN2C, and GluN3 subunits have also been detected within oligodendrocyte processes and myelinating structures in the rat brain (Káradóttir et al., 2005; Salter and Fern, 2005). The observed colocalization of NR1 with NR2C and of NR3A with NR2C within oligodendrocyte processes has been interpreted as indicative of their presence in the same cellular compartments, rather than as coincidental or unrelated staining patterns (Káradóttir et al., 2005). Additionally, co-immunoprecipitation from a purified adult rat brain myelin preparation, lacking detectable astrocyte or neuronal contamination, demonstrated physical association between GluN1, GluN2C, and GluN3A, which has been taken to suggest that these subunits may form a triheteromeric receptor complex in myelin (Burzomato et al., 2010). Support for the existence of such triheteromeric receptors comes from observations of a comparatively weak Mg^2+^ block at resting potential, relative to neuronal NMDA receptors. For instance, Káradóttir et al. (2005) reported that increasing Mg^2+^ concentration from 0 mM to 2 mM only modestly reduced oligodendrocyte NMDA currents (~3–5-fold), whereas a much stronger inhibition was observed in neuronal or cloned receptors (~20–60-fold). This weak block is similar to that observed in cloned NR1/NR2A/NR3A receptors (Burzomato et al., 2010), lending credence to the hypothesis that oligodendrocyte NMDARs possess an atypical subunit composition. In white-matter oligodendrocytes, application of NMDA receptor agonists elicited inward currents that were blocked by the NMDA antagonist D-AP5, but not by TTX, NBQX, strychnine, or bicuculline, suggesting that the observed response is specifically mediated by NMDA receptors (Káradóttir et al., 2005; Salter and Fern, 2005). However, it should be noted that the precise physiological relevance and *in vivo* significance of these findings remain to be fully elucidated.

The involvement of NMDAR in oligodendrocytes has been proposed to encompass at least two processes: participation in axon-glia signaling, purportedly regulating OPC differentiation and myelination (Lundgaard et al., 2013). *In vitro* studies indicate that NMDA signaling can promote OPC maturation, as evidenced by increases in myelin basic protein (MBP) expression, process branching, and the proportion of mature oligodendrocytes. Inhibition of the receptor, either by MK801, the GluN2B subunit-selective antagonist Ro25-6981, or by GluN1 knockdown, reportedly blocks this differentiation (Li et al., 2013). Similarly, in remyelination models, suppression of NMDAR activity appears to delay myelin repair and results in a greater proportion of undifferentiated OPCs and fewer mature oligodendrocytes, observations that have been interpreted as evidence for a role of NMDARs in myelin repair in the adult CNS (Li et al., 2013; Lundgaard et al., 2013). Assessment of a possible mTOR-dependent mechanism, via application of the mTOR inhibitor rapamycin, resulted in decreased numbers of mature oligodendrocytes and lower MBP expression, suggesting an mTOR-associated pathway. Notably, OPCs lacking genetic deletion of the GluN1 subunit in the CNS of both developing and mature mice were able to proliferate normally, re-reached appropriate densities in gray and white matter, and differentiated without observable delay. These OPCs maintained their characteristic physiological and morphological properties and formed synapses with glutamatergic axons despite the absence of NMDAR signaling. Furthermore, expression of calcium-permeable AMPARs was increased in NMDAR-deficient OPCs (De Biase et al., 2011). These findings complicate the interpretation of cell-based data and raise questions about whether NMDAR signaling is truly required for OPC development or whether its primary function instead pertains to the regulation of AMPAR-dependent signaling with neighboring axons, thereby suggesting additional, as yet incompletely understood, roles.

Ketamine is reported to restore myelination in the brains of chronically stressed mice, likely by promoting OPC differentiation into mature oligodendrocytes (Figure 3). Notably, knockdown of myelin-associated oligodendrocytic basic protein blocks the long-lasting behavioral effects of ketamine, as demonstrated by Huang et al. However, it is important to emphasize that this study does not provide evidence directly linking the observed effects to NMDA receptor activity. Therefore, the mechanistic basis of ketamine's influence on myelination and behavior remains somewhat ambiguous, and the involvement of NMDARs in these processes cannot be conclusively established from the available data (Huang et al., 2024).

#### 3.2.3 NMDA receptors in microglia

Real-time PCR analyses have indicated that microglia express GluN1, GluN2A-C, and GluN3 subunits at substantially lower levels than neurons in primary cultures, except for NR2D, which appears unusually elevated. This distinctive NR2D predominance is often interpreted as indicative of a unique microglial subunit composition, though the functional consequences of this profile remain to be fully established (Kaindl et al., 2012). The presence of GluN1, GluN2B, and GluN2D has been demonstrated in cultured murine cortical microglia, as well as in murine brain and spinal cord white matter sections, and in human postmortem brain tissue from both fetuses and adults. Functional studies, such as calcium imaging, suggest that NMDA stimulation can elicit intercellular Ca^2+^ increases in approximately 10% of microglial cells, with these effects being blocked by the NMDAR inhibitor MK801. Patch-clamp electrophysiological recordings in Mg^2+^-containing solution have recorded inward currents (~30 pA) in 5%−10% of microglia upon application of NMDA and D-serine, which were similarly sensitive to MK801 inhibition (Kaindl et al., 2012). It is also noteworthy that both resting and activated microglia exhibit membrane potentials around −40 mV and −45 mV, respectively, which have been considered optimal for NMDAR activation, given that typical neuronal NMDARs are operational at membrane potentials near −40 mV (Boucsein et al., 2000; Kaindl et al., 2012). However, the physiological relevance and prevalence of functional NMDARs in microglia *in vivo* remain subjects of ongoing investigation.

Stimulation of microglial NMDARs with NMDA has been reported to induce an activated state in these cells (Kaindl et al., 2012). In this state, microglia produce reactive oxygen species (ROS) and nitric oxide (NO), thereby increasing oxidative stress (Mander and Brown, 2005). Concurrently, they release pro-inflammatory cytokines such as TNF-α and interleukins, which have the potential to damage neuronal membranes, disrupt cellular function, and ultimately contribute to neuronal cell death. Consequently, neurons exposed to factors secreted by NMDAR-activated microglia exhibit reduced survival. Moreover, it has been suggested that damaged neurons release glutamate, which may further stimulate microglial NMDARs. This is proposed to create a self-perpetuating cycle wherein neuronal injury leads to microglial activation, which in turn exacerbates neuronal damage. Nonetheless, the extent to which these mechanisms operate *in vivo*, and their broader pathophysiological significance, remain areas requiring further clarification and empirical support (Kaindl et al., 2012).

The mechanistic role of ketamine in modulating microglial function in the context of anxiety or depression remains relatively underexplored. In the lipopolysaccharide (LPS)-mediated BV2 cell inflammation model, treatment with varying concentrations of ketamine was reported to attenuate LPS-induced effects, including upregulation of proinflammatory cytokines and enzymes, inhibition of calmodulin-dependent protein kinase II (CaMK II) and its phosphorylation, as well as NF-κB phosphorylation and nuclear translocation. Notably, AP5, another NMDA receptor antagonist, produced effects similar to those of ketamine, suggesting that the observed changes may be attributable to NMDAR inhibition (Lu et al., 2020). The antidepressant and anti-inflammatory effects of ketamine have also been documented in the LPS-induced depressive-like murine model, where ketamine administration (10–20 mg/kg) alleviated despair and anhedonia as assessed by TST and SPT tests. These behavioral improvements were accompanied by reductions in inflammatory CD45highLy6GnegLy6Chigh monocytes in the brain and decreased microglial activation (CD45lowCD11bhigh). The involvement of NMDARs in these effects is further supported by similar outcomes with the NMDA receptor antagonist MK-801 (Arena et al., 2026). Consistent with findings in astrocyte models using CUMS mice, ketamine was shown to reduce microglial activation and the release of pro-inflammatory cytokines (IL-1β, TNF-α) (Wang et al., 2015). Also, in a mouse model of neuropathic pain induced by chronic constriction injury (CCI), S-ketamine (10 mg/kg) was shown to produce sustained analgesic effects by reversing mechanical and thermal hypersensitivity. Mechanistically, CCI increased spinal microglial activation and promoted microglia-mediated phagocytosis of perineuronal nets (PNNs), leading to network destabilization and pain sensitization. S-ketamine reduced microglial activation, suppressed inflammatory cytokines, and decreased engulfment of PNN components, thereby preserving neuronal network integrity. These findings suggest that ketamine's analgesic effects in chronic pain are mediated, at least in part, through NMDA receptor–linked modulation of microglial phagocytic activity and neuroinflammatory signaling (Yang et al., 2025). However, it is important to underscore that the current body of evidence is limited and somewhat heterogeneous; not all studies corroborate these findings. Thus, while a small number of studies suggest that ketamine may exert anti-inflammatory effects through microglia, more focused research is needed to clarify these mechanisms and establish their relevance to anxiety and depression (Figure 3).

## 4 Opioid receptors and evidence for interaction with ketamine

### 4.1 Opioid receptors overview

Opioid receptors, classified as μ (mu), κ (kappa), δ (delta), and nociceptin/orphanin FQ peptide (NOP) receptors, were first characterized in 1973 (Pert and Snyder, 1973; Terenius, 1973; Simon et al., 1973), with their endogenous ligands (enkephalins, endorphins, and dynorphins) identified shortly thereafter (Akil et al., 1976). Opioid receptors belong to the G protein-coupled receptor (GPCR) superfamily and share considerable structural homology, with conserved intron-exon junctions suggesting evolution from a common ancestral gene (Wei and Loh, 2011). All classical opioid receptors couple primarily to inhibitory G proteins (Gi/Go). Upon agonist binding, conformational changes in the receptor activate G protein signaling cascades that lead to: (1) inhibition of adenylyl cyclase, (2) activation of inwardly rectifying potassium channels (GIRKs), (3) inhibition of voltage-gated calcium channels, and (4) modulation of mitogen-activated protein kinase (MAPK) pathways (Allouche et al., 2014; Swingler et al., 2025). These signaling events collectively reduce neuronal excitability and decrease neurotransmitter release.

The four receptor genes, OPRM1 (μ), OPRD1 (δ), OPRK1 (κ), and OPRL1 (NOP), encode the μ opioid receptor (MOR), δ opioid receptor (DOR), κ opioid receptor (KOR), and nociceptin receptor (NOP), respectively (Wei and Loh, 2011). The NOP receptor, while structurally related to classical opioid receptors, is pharmacologically distinct: it does not bind conventional opioid ligands, and its endogenous ligand nociceptin/orphanin FQ exhibits negligible affinity for MOR, DOR, or KOR (Thompson et al., 2012; Zaveri et al., 2013).

More than 20 endogenous opioid peptides have been identified, derived from four precursor proteins: proopiomelanocortin (POMC), proenkephalin (PENK), prodynorphin (PDYN), and prepronociceptin (PNOC) (Al-Eitan et al., 2021). Receptors and peptides are broadly expressed throughout the nervous system, where they control nociceptive pathways (pain sensation), emotional processing, mood and hedonic tone (well-being), stress responses, and reward/motivation circuits (Mansour et al., 1987; Tempel and Zukin, 1987; Mansour et al., 1994). The system also regulates respiratory function, gastrointestinal motility, and endocrine and immune functions (Le Merrer et al., 2009; Dalefield et al., 2022). Genetic polymorphisms in opioid receptor genes have been associated with various neurological and psychiatric conditions (Stoeber et al., 2018).

Opioid receptors undergo dynamic regulation through phosphorylation, desensitization, internalization, and recycling, with these processes differing substantially among receptor subtypes (Allouche et al., 2014). Receptor heterogeneity is further increased by the formation of homodimers, heterodimers, and higher-order oligomers, which can alter pharmacological properties, signaling profiles, and subcellular trafficking (Quirion et al., 2020). The existence of multiple conformational states, influenced by cellular environment and protein-protein interactions, creates functional diversity that complicates drug development but simultaneously offers opportunities for selective therapeutic interventions (Williams et al., 2013; Valentino and Volkow, 2018).

### 4.2 Neuroanatomical distribution and functional organization

Opioid receptors are ubiquitously expressed throughout the central nervous system (CNS) and peripheral nervous system (PNS) structures, including the cortex, limbic regions, basal ganglia, hypothalamus, thalamus, brainstem, and spinal cord; however, they exhibit distinct microarchitectural characteristics despite their overlapping macro-distribution (Gray et al., 2006; Erbs et al., 2015). The MOR and DOR are organized into discrete populations within the forebrain yet co-localize as putative heteromers within subcortical and hindbrain neural circuits (Kareem et al., 2023). The KOR demonstrates a disproportionate enrichment within the NAc, where stress-induced release of dynorphin suppresses dopaminergic activity, positing a potential mechanism underlying anhedonia (Knoll and Carlezon, 2010; Van't Veer and Carlezon, 2013), whereas the DOR is predominantly associated with cortico-limbic networks. Importantly, the distribution of KOR mRNA in humans diverges from that observed in rodent models (George et al., 1994), highlighting the translational limitations inherent to preclinical studies.

### 4.3 Subcellular localization and trafficking

Receptor subtypes exhibit significantly divergent subcellular distribution (Table 1), which has direct implications for signaling outcomes. The MOR is primarily localized within intracellular compartments, particularly in multivesicular bodies designated for degradation, despite the presence of a functionally adequate surface pool that mediates opioid analgesia (Erbs et al., 2015); in contrast, the DOR preferentially resides at the plasma membrane, while the nociceptin/orphanin FQ peptide receptor (NOP) disperses throughout the soma and neuritic processes without membrane confinement (Ozawa et al., 2015). Presynaptic receptor pools demonstrate a resistance to agonist-induced desensitization when compared to postsynaptic receptors, a differential that has direct implications for mechanisms of tolerance (Coutens and Ingram, 2023).

**Table 1:** Subcellular distribution of opioid receptor subtypes.

Receptor	Primary subcellular localization	Primary functions	Anatomical distribution	Key features
MOR	Intracellular; limited plasma membrane	Nociception (pain control), reward, motivation, emotional/cognitive processing, autonomic/sensory modulation	Brain, spinal cord, dorsal horn (lamina II), small DRG neurons, hippocampus, hypothalamus, brainstem nuclei	Substantial localization to multivesicular bodies
DOR	Plasma membrane	Nociception, analgesic tolerance, mood regulation, emotional and cognitive processing	DRGs (medium/large neurons), hippocampus, hypothalamus, brainstem nuclei	Highly regulated trafficking; dynamic surface expression
KOR	Mixed intracellular and membrane	Anti-reward system; mediates stress-induced dysphoria and psychopathology; mood regulation	Nucleus accumbens (presynaptic terminals of DA neurons), various brainstem nuclei	Context-dependent distribution; dynorphin-mediated signaling
NOP	Distributed throughout soma and processes	Modulatory roles (non-opioid receptor)	Brain, spinal cord, dorsal root ganglia	Present in brain, spinal cord, and dorsal root ganglia

The expression of opioid receptors in glial cells remains a topic of considerable debate; presence of MOR in astrocytes and microglia has been reported through immunohistochemical methods (Stiene-Martin et al., 2001; Horvath et al., 2010; Ferrini et al., 2013; Nam et al., 2018), yet concerns regarding antibody specificity and the near-absence of transcript/protein detection via single-cell RNA sequencing (scRNA-seq) and proteomics have undermined these findings (Rangaraju et al., 2018; Maduna et al., 2019). The KOR, however, presents more compelling evidence: microglial expression exhibits anti-inflammatory properties upon activation but is strikingly restricted to specific regions (enriched in the NAc, while sparse in other areas; Saunders et al., 2018), reflecting broader heterogeneity within microglial populations. Oligodendrocytes exhibit developmentally regulated expression of MOR (but not KOR), which governs differentiation and influences morphine-induced cytotoxicity (Hahn et al., 2010). Further resolution awaits spatial transcriptomic and single-cell proteomic investigations across various regions and disease states, paralleling the peripheral roles of opioid receptors in the regulation of respiratory, gastrointestinal, endocrine, and immune functions (Coutens and Ingram, 2023). This widespread distribution enables the opioid system to integrate and modulate various physiological and behavioral responses across multiple organ systems (Figure 4).

**Figure 4:**
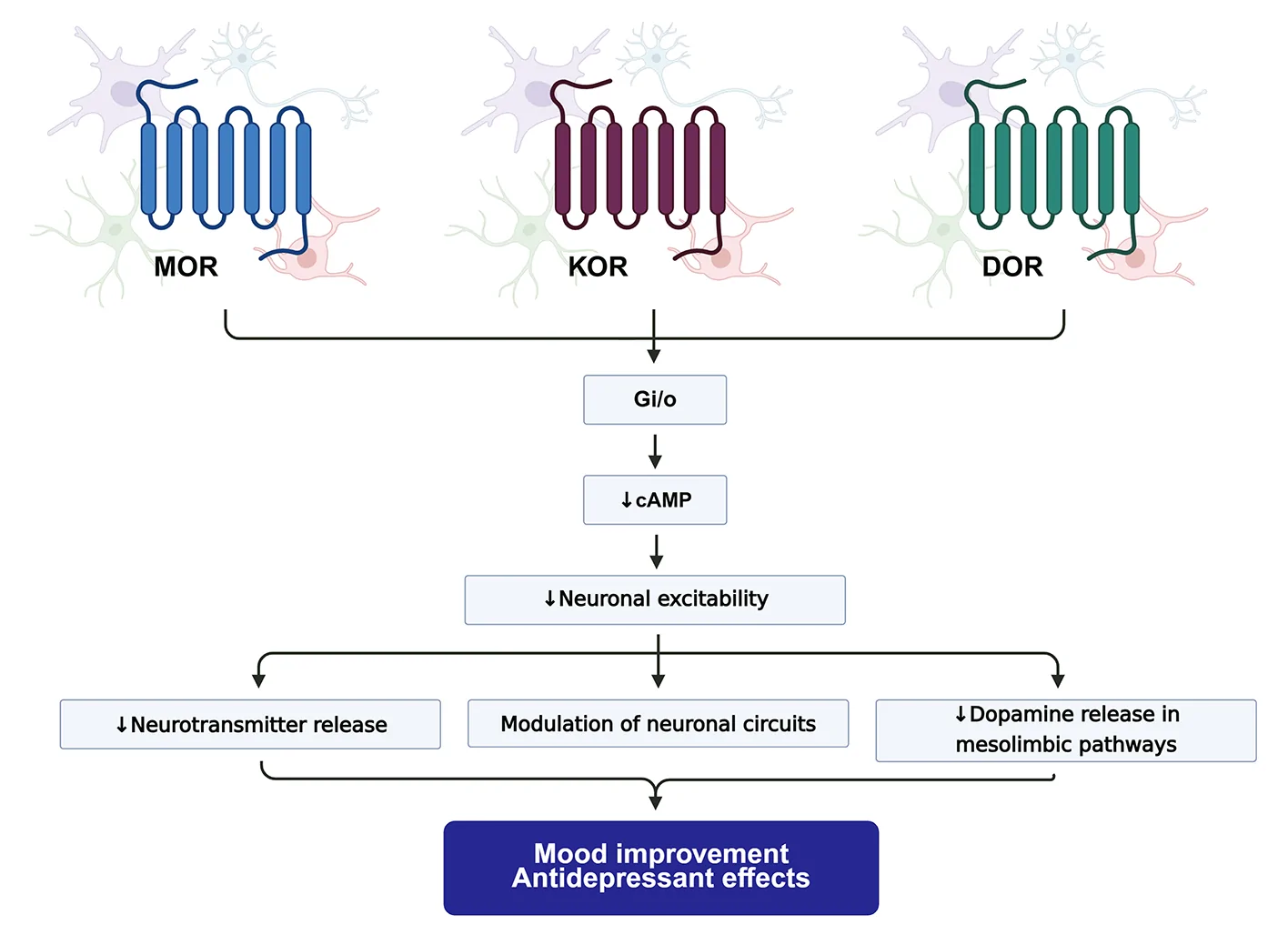
The possible involvement of opioid receptor signaling in the antidepressant effects of ketamine is shown. The schematic opioid MOR, KOR, and DOR receptors, which are present on neurons, astrocytes, microglia, and oligodendrocytes. In neurons, activation of opioid receptors results in Gi/o-dependent signaling, inhibiting adenylyl cyclase, decreasing cAMP production, and modulating neuronal excitability and neurotransmitter release. These effects could affect neuronal circuit activity, such as that of the mesolimbic dopaminergic system, and thus may contribute to the antidepressant action of ketamine. Opioid receptors are also found on glial cells; however, the specific way in which they contribute to the antidepressant and neuroplastic effects induced by ketamine is not well characterized. Created in BioRender. Sulej, Z. (2026).

### 4.4 Evidence for ketamine-opioid receptor interactions

Ketamine's interaction with opioid receptors has been documented through multiple experimental approaches, though the nature and functional significance of these interactions remain partially controversial (Coutens and Ingram, 2023). Importantly, ketamine binds to MOR and KOR with micromolar affinity, exhibiting slightly lower potency but higher intrinsic efficacy at KOR compared to MOR (Hirota et al., 1999; Jiang et al., 2026).

Critical pharmacological differences are present between ketamine enantiomers; S-ketamine demonstrates 2–3-fold greater potency at opioid receptors compared to racemic ketamine, alongside its approximately 4-fold higher affinity for NMDA receptors relative to the racemic mixture (Stoeber et al., 2018). In contrast, R-ketamine shows reduced potency at both NMDA and opioid receptors yet paradoxically exhibits antidepressant-related actions with reduced dissociative effects and lower abuse liability compared to S-ketamine (Zanos et al., 2018). The primary ketamine metabolite HNK demonstrates minimal opioid receptor activity, which may contribute to its lower abuse potential and therapeutic promise as a safer agent (Levinstein et al., 2026). The proposed contribution of MOR, KOR, and DOR signaling in neurons and glial cells to ketamine's antidepressant effects is summarized in Figure 4.

A major limitation in the existing literature is the frequent failure to differentiate among opioid receptor subtypes when investigating ketamine's mechanisms of action. Some preclinical studies have suggested that KOR signaling may contribute to ketamine's cognitive and behavioral effects (Nemeth et al., 2010), though the relative contributions of MOR, DOR, and KOR to therapeutic vs. adverse effects remain incompletely characterized.

#### 4.4.1 Clinical evidence: naltrexone blockade studies

The most compelling clinical evidence for opioid system involvement in ketamine's antidepressant effects comes from naltrexone (NTX) blockade studies. Williams et al. (2018) conducted a randomized, placebo-controlled trial demonstrating that pretreatment with NTX (50 mg oral, administered 45 min before infusion) significantly attenuated both the antidepressant and anti-suicidal effects of intravenous ketamine (0.5 mg/kg) in adults with TRD. Critically, NTX did not block ketamine's dissociative effects (measured by the Clinician-Administered Dissociative States Scale), indicating that dissociative symptoms and antidepressant efficacy are mediated by distinct neurobiological mechanisms. This finding challenged the prevailing hypothesis that ketamine's therapeutic effects are mediated exclusively through NMDA receptor antagonism and provided the first direct human evidence that opioid receptor activation is necessary for ketamine's rapid antidepressant action.

However, the evidence is not entirely consistent across studies. At least one investigation failed to replicate the NTX blockade of ketamine's antidepressant effects (Pacheco et al., 2014), suggesting that opioid receptor contributions may be context-dependent or that compensatory mechanisms can maintain therapeutic efficacy when opioid signaling is pharmacologically blocked. Methodological differences, including ketamine dosing regimens, NTX administration timing, patient selection criteria (TRD vs. MDD without treatment resistance), and outcome measures, may partially account for these discrepancies. Notably, patients with TRD appear to show stronger opioid system involvement compared to those with less refractory depression, potentially reflecting underlying neurobiological differences (e.g., in GABAergic function) between these populations (Lu et al., 2026).

#### 4.4.2 Preclinical evidence: rodent models

Preclinical studies have produced mixed results regarding opioid system involvement in ketamine's behavioral effects. In congenitally learned helplessness rats, NTX coadministration reduced ketamine's antidepressant-like effects: compared to ketamine alone, the NTX combination increased immobility time in the forced swim test, prevented improvements in reward-seeking behavior, and blocked ketamine-induced reductions in LHb neuronal activity, a brain region hyperactive in depression. Similarly, the peptide opioid antagonist CTOP (D-Phe-Cys-Tyr-D-Trp-Arg-Thr-Pen-Thr-NH_2_) inhibited ketamine-related decreases in LHb activity (Klein et al., 2020).

Conversely, other studies have failed to find significant opioid involvement. In multiple stress-induced mouse models, NTX coadministration did not significantly attenuate ketamine's antidepressant-like effects in the forced swim test, tail suspension test, or sucrose preference test (Pomrenze et al., 2025; Zhang and Hashimoto, 2019). Similarly, in a zebrafish model of chronic unpredictable stress, although proteomic analyses revealed that ketamine normalized stress-related dysregulation of opioid signaling pathway proteins (including RICTOR, PER2, and CABP1), these findings suggest modulation rather than direct dependence of ketamine's effects on opioid signaling (Reddy et al., 2021).

These divergent results may reflect: (1) differences in depression model validity and underlying pathophysiology, (2) species-specific variations in opioid system organization, (3) distinct pharmacological properties of racemic ketamine vs. individual enantiomers, and (4) differential roles of MOR, DOR, and KOR in various aspects of depressive-like behavior and antidepressant response (Lu et al., 2026).

#### 4.4.3 Mechanistic interpretations

Evidence suggests that ketamine does not function as a classical opioid receptor agonist but rather modulates opioid receptor signaling through indirect mechanisms or allosteric modulation (Levinstein et al., 2025a). Given ketamine's relatively low binding affinity for opioid receptors at clinically relevant antidepressant doses (submicromolar to low micromolar plasma concentrations), direct competitive agonism appears unlikely to fully account for its therapeutic effects (Hirota et al., 1999). Alternative mechanisms may include: (1) positive allosteric modulation enhancing the effects of endogenous opioid peptides, (2) indirect enhancement of endogenous opioid release through effects on glutamatergic or other neurotransmitter systems, or (3) modulation of opioid receptor trafficking or signaling efficacy (Gupta et al., 2025).

Opioid receptor engagement by ketamine may influence dopamine release in reward-related circuits, potentially contributing to the drug's mood-elevating and, in the case of S-ketamine, reinforcing properties (Di Ianni et al., 2024). This pharmacological profile positions S-ketamine as the primary contributor to abuse liability, while R-ketamine and metabolites such as HNK represent potentially safer therapeutic alternatives with reduced opioid receptor activity (Yang et al., 2015; Zanos et al., 2018).

Paradoxically, despite ketamine's own potential for misuse, it has shown promise in the treatment of substance use disorders, including opioid, alcohol, and cocaine dependence, where it may attenuate withdrawal symptoms and reduce drug craving, potentially serving as an adjunct therapy during early recovery phases (Jones et al., 2018; Walsh et al., 2022).

The available evidence supports a model in which the opioid system functions as a necessary but not sufficient mediator of ketamine's antidepressant actions (Williams et al., 2018; Klein et al., 2020) (see Figure 4). This characterization positions opioid receptor engagement as a modulator rather than the sole driver of therapeutic effects. One proposed mechanistic framework suggests that transient, partial activation of opioid receptors by ketamine contributes to rapid anti-suicidal effects, whereas sustained, full opioid receptor agonism (as with classical opioids) is associated with increased suicide risk (Schatzberg, 2014; Williams et al., 2018). This dose-response and temporal relationship may explain how ketamine achieves therapeutic benefits while avoiding the adverse neuropsychiatric consequences of chronic opioid agonism.

## 5 Sigma 1 receptors and evidence for interaction with ketamine

### 5.1 Sigma 1 receptor overview

In addition to the interactions described above, ketamine has been reported to interact with sigma receptors, including the sigma-1 receptor (S1R), a potential modulator of ketamine-induced neuroplasticity. S1R, encoded by the *SIGMAR1* gene, is a ligand-regulated transmembrane protein that functions mainly as an intracellular molecular chaperone and signaling modulator rather than a conventional cell-surface receptor. S1R is widely distributed throughout the CNS and is involved in the regulation of intracellular Ca^2+^ homeostasis, endoplasmic reticulum (ER) stress responses, mitochondrial function, neuronal excitability, and cell survival (Ryskamp et al., 2019; Wu et al., 2021; Malar et al., 2023).

High-resolution crystallographic studies revealed a trimeric architecture of S1R, where each protomer possesses a single N-terminal transmembrane helix, a cupin-like β-barrel domain with a ligand-binding site, and a C-terminal V-shaped two-helix bundle forming a lid over the binding pocket. The ligand-binding pocket is highly conserved and mostly hydrophobic, with Glu172 involved in ligand recognition and Asp126 organizing the binding site (Ryskamp et al., 2019; Meng et al., 2022). S1R exists in a dynamic equilibrium among monomers, dimers, trimers, and higher-order oligomers rather than only the trimeric state. Ligand binding can modulate this equilibrium, potentially providing a mechanism for S1R ligands to regulate receptor function (Chu and Ruoho, 2016; Ryskamp et al., 2019).

Studies have shown widespread S1R distribution in rodent and guinea pig brains, including the cortex, hippocampus, striatum, thalamus, hypothalamus, substantia nigra, cerebellum, and brainstem nuclei (Penke et al., 2018; Liu Q. et al., 2023). Cell-type-specific studies in mice identified S1R in excitatory neurons, parvalbumin- and somatostatin-positive interneurons, astrocytes, microglia, oligodendrocyte precursor cells, oligodendrocytes, Purkinje neurons, and Bergmann glia across multiple brain regions (Liu Q. et al., 2023). *In vitro* studies also demonstrated S1R expression in oligodendrocytes, oligodendrocyte progenitor cells (OPCs), and microglia (Su et al., 2016; Ooi et al., 2021).

In the human post-mortem brain, *SIGMAR1* mRNA was detected in the temporal cortex, particularly within deeper cortical layers, and in the hippocampus (Kitaichi et al., 2000). *In vivo* PET imaging using the S1R-selective radioligand [^11^C]SA4503 has also demonstrated widespread S1R availability throughout the living human brain (Sakata et al., 2007).

At the subcellular level, S1R is mainly localized to the endoplasmic reticulum (ER) and enriched at mitochondria-associated ER membranes (MAMs), specialized contact sites coordinating ER–mitochondrial communication, Ca^2+^ transfer, and cellular bioenergetics (Kourrich et al., 2012; Ryskamp et al., 2019; Lachance et al., 2023). S1R associates with the ER-resident chaperone BiP/GRP78. ER Ca^2+^ depletion, ER stress, or stimulation by S1R agonists promote dissociation of this complex, increasing S1R availability to interact with client proteins (Hayashi and Su, 2007; Ryskamp et al., 2019). One well-characterized target at MAMs is IP_3_R3, which S1R stabilizes after dissociation from BiP, facilitating sustained Ca^2+^ transfer from the ER to mitochondria and supporting mitochondrial function and energy metabolism (Hayashi and Su, 2007; Ryskamp et al., 2019; Lachance et al., 2023). Through this mechanism, S1R contributes to intracellular Ca^2+^ homeostasis, especially under cellular stress (Ryskamp et al., 2019; Shi et al., 2021).

S1R also acts as an adaptive modulator of ER stress and modulate major unfolded protein response (UPR) pathways, including PERK, IRE1α, and ATF6 (Penke et al., 2018; Shi et al., 2021). Specifically, S1R can stabilize IRE1α and support adaptive IRE1α-XBP1 signaling while limiting prolonged stress responses linked to CHOP-mediated toxicity (Hayashi, 2019; Aishwarya et al., 2021).

S1R also modulates store-operated Ca^2+^ entry (SOCE) by interacting with STIM1, reducing STIM1–Orai1 coupling and limiting extracellular Ca^2+^ influx after ER Ca^2+^ depletion (Srivats et al., 2016). Furthermore, S1R regulates several voltage-gated Ca^2+^, K^+^, and Na^+^ channels, thereby contributing to neuronal excitability and the control of neurotransmitter release (Su et al., 2016; Yang et al., 2019).

In neurons, S1R can modulate NMDAR trafficking and signaling, potentially enhancing physiological NMDAR-dependent synaptic signaling while limiting excitotoxic downstream pathways under pathological conditions (Shi et al., 2021; Malar et al., 2023) S1R can additionally interact with GPCR-related signaling systems and modulate downstream kinase pathways, including ERK1/2, Akt, and CREB (Chu and Ruoho, 2016; Yang et al., 2019; Shi et al., 2021).

In glia cells, S1R has been implicated in the regulation of astrocytic reactivity and suppresses pro-inflammatory microglial responses (Weng et al., 2017; Jia et al., 2018; Ooi et al., 2021) and in oligodendrocyte differentiation and myelin-related processes in OPCs and mature oligodendrocytes (Hayashi and Su, 2004; Liu Q. et al., 2023).

Overall, S1R acts as an intracellular regulatory chaperone that integrates Ca^2+^ homeostasis, ER–mitochondrial communication, neuronal excitability, and cellular stress responses, contributing to neuroprotection, synaptic plasticity, and the regulation of both neuronal and glial function (Malar et al., 2023; Ryskamp et al., 2019).

### 5.2 Ketamine-sigma 1 receptor interactions

Racemic ketamine has been reported to bind sigma receptors, including sigma-1 receptor (S1R), with micromolar affinity, although the extent and functional relevance of this direct interaction appear to depend on the experimental binding assay (Robson et al., 2012; Zanos et al., 2018). Moreover, (R)-ketamine has been reported to display higher affinity for S1R than (S)-ketamine, although the significance of this stereoselectivity for ketamine's antidepressant action remains unclear (Zanos et al., 2018). Overall, these findings indicate that direct interaction with S1R is possible but substantially weaker than that observed for classical high-affinity S1R ligands.

Functional evidence suggests that S1R may contribute more prominently to ketamine-induced neuroplasticity than to its immediate antidepressant-like behavioral effects (see Figure 5). In PC12 cells, ketamine enhanced nerve growth factor (NGF)-induced neurite outgrowth, and this effect was attenuated by the S1R antagonist NE-100, implicating sigma receptor signaling in ketamine-induced neuronal remodeling (Robson et al., 2012). Consistent with a functional effect on S1R, ketamine also reduced the association between S1R and the endoplasmic reticulum chaperone BiP/GRP78 in S1R-overexpressing HEK293T cells in a concentration- and time-dependent manner (Ma et al., 2024). Because dissociation of S1R from BiP is commonly associated with functional activation of the receptor, these observations support the possibility that ketamine can modulate S1R signaling even when its direct receptor affinity is relatively weak.

**Figure 5:**
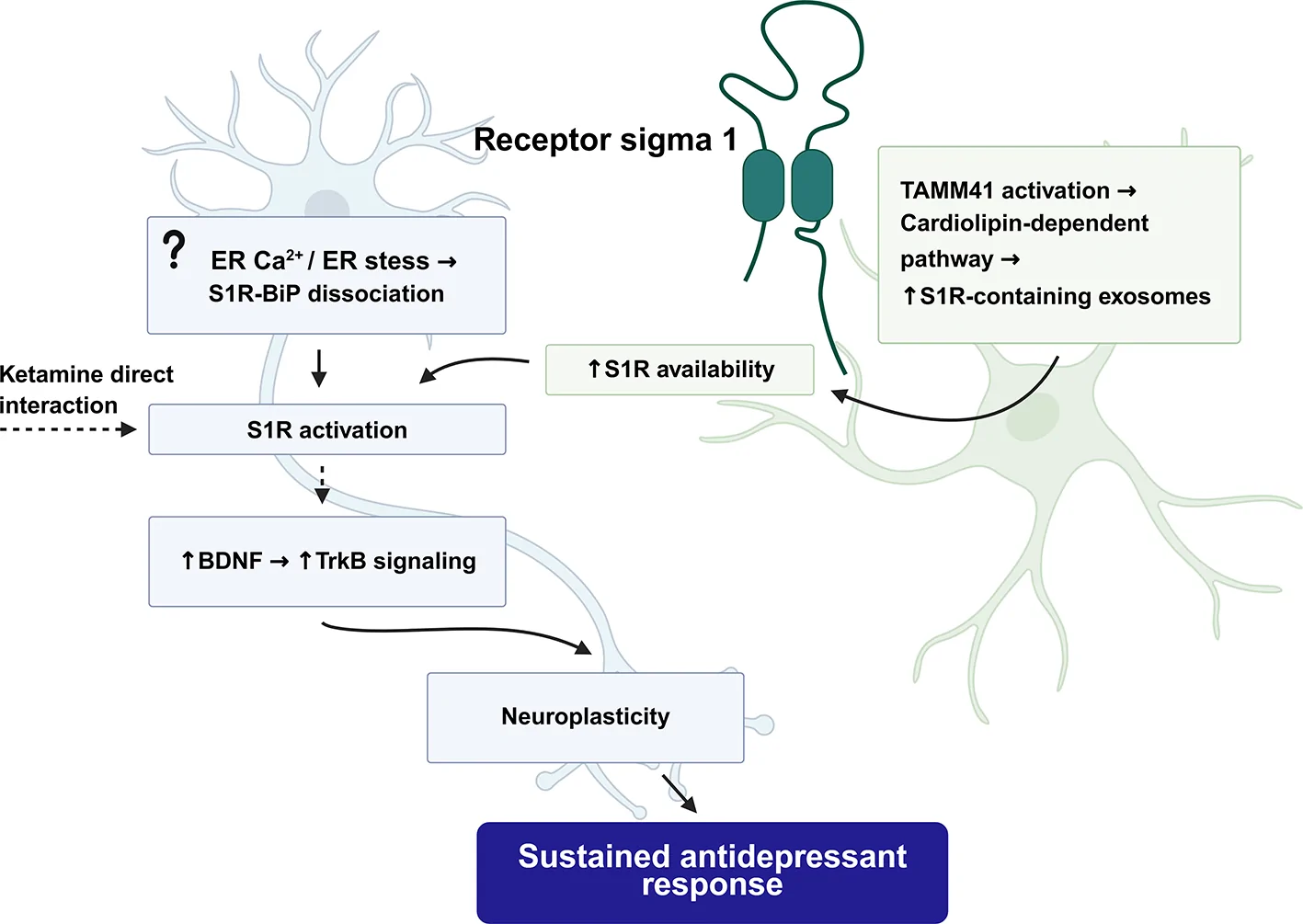
A proposed role of sigma-1 receptor signaling in signaling in ketamine-induced neuroplasticity and sustained antidepressant-like effects. Ketamine might affect the S1R both by weakly interacting with the receptor and through indirect mechanisms. An indirect pathway may involve changes in intracellular Ca^2+^ homeostasis and/or endoplasmic reticulum (ER) stress, which could lead to the dissociation of S1R from the chaperone BiP/GRP78, thereby enhancing S1R activity. Once activated, the S1R may boost BDNF-dependent TrkB signaling and neuronal plasticity, thereby contributing to sustained antidepressant-like effects. At the same time, ketamine could activate astrocytic TAMM41 and promote increased exosomal transfer of S1R from astrocytes to neurons, a process dependent on cardiolipin, thereby increasing the amount of S1R available in neurons and supporting the same downstream neuroplastic pathway. Arrows that are solid show relationships that have been established by experiment, while dashed arrows and question marks refer to mechanisms that are proposed or still unresolved. Created in BioRender. Sulej, Z. (2026).

However, pretreatment with the sigma receptor antagonists NE-100 or BD1047 did not prevent the acute antidepressant-like effects of ketamine in the mouse forced swim test (Robson et al., 2012). These findings suggest that S1R signaling is not obligatory for the acute behavioral response to ketamine in this experimental paradigm and may instead be more relevant to plasticity-related and sustained effects. Supporting this interpretation, pharmacological blockade of S1R with BD1047 attenuated ketamine-induced structural and functional restoration of mPFC pyramidal neurons, whereas pharmacological activation of S1R with SA4503 enhanced the sustained antidepressant-like effects of ketamine in mice (Ma et al., 2024).

The mechanism through which ketamine activates S1R remains unresolved. Ma et al. (2024) found that ketamine produced little displacement of the S1R-selective radioligand ^3^H-pentazocine, despite inducing a reduction in S1R–BiP association. These findings led the authors to propose that ketamine may activate S1R predominantly through an indirect mechanism rather than through high-affinity binding to the classical S1R ligand-binding site. One possibility is that ketamine-induced alterations in intracellular Ca^2+^ homeostasis and endoplasmic reticulum stress modify the interaction between S1R and BiP/GRP78, conditions known to influence S1R activation and chaperone function (Hayashi, 2018; Ryskamp et al., 2019; Ma et al., 2024). However, a direct causal sequence linking ketamine-induced Ca^2+^ alterations or ER stress to S1R–BiP dissociation has not yet been established, and the upstream mechanism responsible for ketamine-induced S1R activation therefore remains unclear.

A major downstream component of S1R-dependent ketamine signaling appears to involve BDNF. Ketamine increased BDNF expression, whereas S1R knockdown attenuated ketamine-induced BDNF upregulation and associated neuroplastic effects (Ma et al., 2024). Pharmacological manipulation of S1R signaling in the mPFC also modified the sustained behavioral and neuroplastic responses to ketamine. Notably, BDNF knockdown prevented the enhancement of ketamine's sustained antidepressant-like effect produced by the S1R agonist SA4503 (Ma et al., 2024). These findings position S1R upstream of BDNF signaling within this experimental model and suggest that S1R activation facilitates ketamine-induced structural and functional restoration of the mPFC pyramidal neurons. This mechanism may converge with the broader BDNF–TrkB-dependent plasticity pathways that have been implicated in ketamine-induced synaptic remodeling and sustained antidepressant responses (Zanos et al., 2018; Ma et al., 2024).

Recent evidence extended the potential role of S1R in astrocyte–neuron communication; ketamine was reported to activate the mitochondrial protein TAMM41 in astrocytes, promoting a cardiolipin-dependent increase in the exosomal transfer of astrocytic S1R to neurons (Guo et al., 2026). Conditional deletion of astrocytic TAMM41 disrupted this pathway and attenuated the sustained antidepressant-like effects of ketamine. Moreover, engineered exosomes loaded with S1R mRNA were capable of delivering S1R to neurons in S1R-knockout mice and producing antidepressant-like behavioral effects (Guo et al., 2026). These observations suggest that ketamine may regulate not only S1R activity within individual cells but also the intercellular availability of S1R, providing a potential astrocyte-dependent mechanism contributing to sustained neuronal plasticity.

Overall, S1R is a modulatory component of ketamine-induced neuroplasticity and sustained antidepressant-like responses rather than an obligatory primary mediator of its acute antidepressant-like effects (Robson et al., 2012; Zanos et al., 2018; Ma et al., 2024). Ketamine may influence S1R through a combination of weak direct receptor interactions and indirect mechanisms that alter S1R activation, chaperone function, or cellular availability. S1R-dependent enhancement of BDNF signaling and astrocyte-to-neuron transfer of S1R may contribute to the restoration of neuronal structure and function and thereby support the persistence of ketamine-induced antidepressant effects (Ma et al., 2024; Guo et al., 2026). Nevertheless, although S1R is known to regulate Ca^2+^ homeostasis, ER stress responses, mitochondrial function, and receptor signaling, direct evidence demonstrating that these individual S1R-dependent pathways are necessary for ketamine's antidepressant effects remains limited.

## 6 Integration and future directions

### 6.1 Toward a multi-receptor convergence model of antidepressant ketamine action

The mechanisms reviewed here remain largely hypothetical pending clinical verification, are not mutually exclusive, and may in some cases converge on shared pathways. We therefore propose a multi-receptor, cell-type-specific perspective not as an established model but as a provisional framework for organizing and prioritizing these hypotheses for future testing. The antagonism of NMDARs on tonically active GABAergic interneurons precipitates the response by instigating cortical disinhibition, a transient increase in glutamate levels, and subsequent activation of AMPARs. This biochemical cascade culminates in BDNF–TrkB–mTORC1 signaling, alongside S1R-chaperoned intracellular calcium stabilization underpins intermediate synaptogenesis, and glial homeostatic mechanisms, including the exosomal transfer of S1R, which is instrumental in facilitating synaptic protein synthesis and the maturation of dendritic spines. Importantly, ketamine and its metabolites interact with TrkB directly at its transmembrane domain, thereby stabilizing an active conformation independently of NMDAR inhibition a dual mechanism that may elucidate the rapidity and resilience of its effects on synaptic plasticity in contrast to traditional antidepressants, which seemingly engage TrkB at a more protracted pace.

Glial cells provide the homeostatic support for neuronal response and appear essential in sustaining newly established synapses, albeit most of these effects emerge as secondary responses to neuronal signaling rather than through direct blockade of NMDARs by astrocytes. Additionally, the modulation of microglial activity characterized by reduced release of pro-inflammatory cytokines and normalization of activation states may further contribute to this process, particularly within subtypes of depression characterized by inflammation.

The observation implies that the involvement of opioids may be contextually dependent, likely influenced by the severity of treatment resistance and the integrity of reward circuits, rather than constituting a universal mechanism.

A plausible temporal model delineates all systems in a hierarchical manner: NMDAR-mediated glutamate release and opioid receptor activation facilitate the acute response; BDNF–TrkB–mTORC1 signaling underpins intermediate synaptogenesis and glial homeostatic mechanisms ultimately consolidate and maintain synaptic remodeling over an extended period. This conceptual framework aligns with the clinical time-course of ketamine's effects manifesting within hours, gradually subsiding over a duration of 1–2 weeks.

### 6.2 Key knowledge gaps

Which neuronal and glial populations are essential vs. sufficient for particular facets of the response remains largely ambiguous and warrants investigation employing conditional knockout strategies, chemogenetic techniques, and cell-type-specific imaging methodologies. The function of astrocytic NMDARs *in vivo* is predominantly inferred from indirect evidence; direct experimentation through astrocyte-specific receptor deletion is imperative to ascertain whether astrocytic NMDARs serve as a primary target or function as a downstream signaling node. More broadly, the contribution of glial mechanisms that may occur independently of direct NMDAR blockade also remains insufficiently characterized. Determining whether such glial responses contribute causally to antidepressant efficacy, represent secondary consequences of altered neuronal activity, or arise through receptor-independent actions of ketamine will require further cell-type-specific investigation. The specificity of opioid receptor subtypes is complicated by dependence on non-selective antagonists, whereas the role of MOR, KOR, and DOR in reward processing and mood regulation is antagonistic, thus necessitating subtype-selective pharmacological approaches to elucidate their individual contributions.

Mechanisms specific to enantiomers and metabolites also require further elucidation. R-ketamine and HNK demonstrate diminished engagement with NMDARs and opioid receptors yet preserve antidepressant-like efficacy in certain models, thereby challenging the presumption that NMDAR antagonism is essential for sustained therapeutic effect. Finally, the crosstalk between NMDARs and opioid receptors mediated by shared downstream effectors (such as CaMKII, ERK, and CREB) or reciprocal receptor trafficking remains significantly underexplored and may account for some of the variability observed in blockade studies.

### 6.3 Clinical and translational implications

Recognition of the biological and clinical heterogeneity of depression has prompted increasing differentiation among depressive subtypes, biological endotypes, symptom dimensions, and clinically relevant comorbidity profiles. While the clinical testing of ketamine has primarily focused on TRD, recent research is expanding to include diagnostically and phenotypically more diverse patient populations (Matveychuk et al., 2020; McGrath et al., 2022; Freudenberg et al., 2025). This trend is evident in the ketamine studies currently registered, as summarized in Figure 2. Of the 32 studies reviewed, 24 (75%) enroll patients with depressive disorders, and a significant proportion also investigate clinically relevant comorbidities or distinct symptom profiles. Among these depression-related studies, approximately 25% include PTSD, 25% substance use disorders, 20.8% chronic pain, 20.8% suicidal ideation, and 16.7% anxiety. Bipolar disorder is represented in approximately 20.8%, and OCD in 8.3%. These overlapping categories illustrate the increasing focus on ketamine's effects in clinically complex populations with depression and co-occurring psychiatric, behavioral, or somatic conditions.

Patient heterogeneity may influence ketamine's mechanisms of action. The relative contributions of glutamatergic, neurotrophic, glial or inflammatory, and opioid-related pathways likely vary across individuals. Rather than exerting its effects through a single pathway, ketamine appears to engage multiple overlapping mechanisms, with the most relevant pathways determined by the neurobiological characteristics of each depressive subtype (Zanos et al., 2018; Adzic et al., 2023; Lu et al., 2026). In TRD, which affects about 30% of individuals with MDD and is linked to glutamatergic dysregulation and impaired excitatory-inhibitory balance, mechanisms such as ketamine-induced NMDAR blockade, cortical disinhibition, increased glutamate release, and subsequent AMPAR recruitment may be especially important (Matveychuk et al., 2020; McGrath et al., 2022; Freudenberg et al., 2025). Human imaging studies support this pathway, showing altered AMPAR distribution in TRD and region-specific AMPAR changes after ketamine treatment that correlate with clinical improvement (Nakajima et al., 2026). These findings suggest ketamine may be especially effective in patients with disrupted excitatory-inhibitory balance. However, prospective evidence that baseline abnormalities in glutamatergic signaling or glutamate-GABA balance can reliably identify ketamine responders remains limited (Matveychuk et al., 2020; Freudenberg et al., 2025; Nakajima et al., 2026).

A distinct biological context may be relevant for depressive phenotypes marked by chronic stress exposure, impaired stress adaptation, and neuroplasticity deficits. In these patients, restoring brain BDNF/TrkB/mTORC1 signaling may be a critical part of ketamine's mechanism (Lin et al., 2021; Pardossi et al., 2024; Krystal et al., 2024). Experimental disruption of BDNF or TrkB signaling significantly reduces ketamine-induced synaptic and behavioral effects, highlighting this pathway's importance for ketamine-related neuroplasticity (Lin et al., 2021; Pardossi et al., 2024). mTORC1 mainly contributes to consolidating and maintaining these synaptic changes rather than initiating the rapid antidepressant response (Pardossi et al., 2024; Krystal et al., 2024). The BDNF Val66Met polymorphism and peripheral BDNF concentrations have been studied as potential indicators of individual variability in ketamine response. However, associations between Val66Met and clinical outcomes have been inconsistent, and neither this genetic variant nor peripheral neurotrophic-factor levels currently offer strong predictive value for treatment selection (Matveychuk et al., 2020). Therefore, while restoring BDNF–TrkB-dependent plasticity may be especially relevant to stress-related or low-plasticity depressive phenotypes, direct evidence for a discrete ketamine-responsive endotype remains indirect.

Glial and immune-related mechanisms may be relevant in patients with inflammatory or immune-associated depression phenotypes (Hassamal, 2023). Along with evidence that ketamine can reduce microglial activation and pro-inflammatory signaling, these findings provide a strong biological rationale for increased engagement of glial mechanisms in patients exhibiting significant immune activation (Yao et al., 2022; Wen et al., 2024) Importantly, inflammatory markers such as C-reactive protein (CRP), interleukin-6 (IL-6), and other cytokines are readily measurable in clinical practice, offering practical tools to monitor inflammation and potentially guide personalized treatment strategies based on the patient's immune status. Nevertheless, most evidence directly linking glial mechanisms to ketamine efficacy remains preclinical, and preferential engagement of astrocytic or microglial pathways in prospectively defined inflammatory depression endotypes has not yet been demonstrated (Zanos et al., 2018; Mariani et al., 2022).

Involvement of the opioid system may contribute to interindividual variability in ketamine response, although its association with specific depressive phenotypes remains not fully clear. The most robust clinical evidence for opioid involvement is provided by a randomized crossover study in which pretreatment with naltrexone reduced ketamine's antidepressant effects in patients with TRD, without similarly affecting its dissociative effects (Williams et al., 2018). In the recent study, in patients with MDD and comorbid alcohol use disorder, NTX did not eliminate ketamine's antidepressant effects, though differences in patient characteristics and study design complicate direct comparisons with TRD cohorts (Yoon et al., 2025). Additionally, a recent systematic review also found mixed effects of opioid antagonism across clinical and preclinical studies, rather than supporting an obligatory opioid mechanism for ketamine (Lu et al., 2026). Reported inconsistencies indicate that opioid-system involvement may depend on a plethora of factors such as treatment resistance, comorbidity, reward-circuit function, ketamine formulation, antagonist exposure, or timing of administration (Lu et al., 2026).

### 6.4 Implications for drug development

This convergence model posits several rational strategies for the development of next-generation antidepressants: (1) direct modulators of TrkB that replicate the plasticity effects of ketamine without the dissociative properties associated with NMDAR inhibition; (2) HNK and related metabolites, contingent upon the delineation of their pharmacokinetics and independence from NMDAR activity in human subjects; (3) agents targeting glial cells, such as Kir4.1 inhibitors (e.g., VU0134992, Lys05, JX3212) or GLT-1 enhancers, which may elicit antidepressant effects through astrocytic rather than neuronal mechanisms; (4) subtype-selective or biased opioid ligands that differentiate therapeutic effects from dysphoric or reinforcing outcomes; and (5) combinatorial strategies that pair low-dose ketamine with adjunctive agents (such as anti-inflammatory compounds or AMPAkines) to enhance therapeutic efficacy while mitigating dosing frequency and adverse effects.

### 6.5 Conclusion

The rapid antidepressant effects of ketamine arise from a coordinated interplay among NMDAR-mediated disinhibition, BDNF-TrkB-mTORC1-dependent plasticity, glial homeostatic support, and opioid receptor modulation. Importantly, the relative contribution of each pathway likely differs across the heterogeneous spectrum of clinical depression. For example, patients with inflammatory or immune-driven depression may benefit most from glial and microglial pathway engagement, while patients with pronounced anhedonia or reward circuit dysfunction may rely more on opioid-mediated mechanisms. In turn, TRD, characterized by altered glutamatergic neurotransmission, may be particularly responsive to interventions targeting NMDAR and AMPAR signaling.

This mechanistic heterogeneity provides a framework for interpreting conflicting clinical findings, such as the variable impact of NTX on ketamine's efficacy. The opioid system may be a modulator in some endotypes (e.g., severe, reward-deficient, or treatment-resistant cases), explaining why NTX blockade does not uniformly abolish antidepressant effects. Such variability underscores the need for biomarker-guided patient stratification in future trials.

Translationally, this multi-receptor convergence model supports the development of precision psychiatry approaches. Biomarkers such as inflammatory markers (CRP, IL-6), neuroimaging, and genetic variants (OPRM1, BDNF Val66Met, GRIN2B) could guide individualized treatment selection. Next-generation antidepressants may include TrkB modulators, glial-targeted agents, or subtype-selective opioid ligands, alone or in rational combinations. Ultimately, integrating mechanistic insights with clinical endotyping will enable more effective, personalized interventions and help resolve current ambiguities in rapid-acting antidepressant research.

## Glossary

4E-BP1, eukaryotic translation initiation factor 4E-binding protein 1; 5-HT, 5-hydroxytryptamine (serotonin); 5-HTTLPR, serotonin-transporter-linked polymorphic region; A1R, Adenosine A1 receptor; A2AR, Adenosine A2A receptor; ACC, anterior cingulate cortex; ADP, adenosine diphosphate; Akt, protein kinase B; AMPA, α-Amino-3-hydroxy-5-methyl-4-isoxazolepropionic acid; AMPAR, α-Amino-3-hydroxy-5-methyl-4-isoxazolepropionic acid receptor; ANLS, astrocyte-neuron lactate shuttle; AQP4, aquaporin 4; ATF6, activating transcription factor 6; ATP, adenosine triphosphate; BDNF, brain-derived neurotrophic factor; BiP, binding immunoglobulin protein; BP, bipolar disorder; CaMKII, calcium/calmodulin-dependent protein kinase II; Camp, cyclic adenosine monophosphate; CCI, chronic constriction injury; CNS, central nervous system; CREB, cAMP response element-binding protein; CRP, C-reactive protein; CUMS, Chronic unpredictable mild stress; Cx43, Connexin 43; DA, dopamine; DALYs, disability-adjusted life years; DAPK1, death-associated protein kinase 1; DOR, δ-Opioid receptor; DRG, dorsal root ganglion; EAAT2, excitatory amino acid transporter 2; ECT, electroconvulsive therapy; eEF2, eukaryotic elongation factor 2; eEF2K, eukaryotic elongation factor 2 kinase; EGFP, enhanced green fluorescent protein; ENT1, equilibrative nucleoside transporter 1; ENT2, equilibrative nucleoside transporter 2; ER, endoplasmic reticulum; ERK, extracellular signal-regulated kinase; FDA, food and drug administration; GABA, γ-aminobutyric acid; GIRK, G protein-gated inwardly rectifying potassium channel; GLT-1, glutamate transporter 1; GPCR, G protein-coupled receptor; GRP78, 78-kDa glucose-regulated protein; GS, glutamine synthetase; GSK-3β, glycogen synthase kinase 3 beta; HNK, (2R,6R)-hydroxynorketamine; HPA, hypothalamic-pituitary-adrenal; HPC, homeostatic property cluster; IL-1β, interleukin-1 beta; IL-6, interleukin-6; IP_3_, inositol 1,4,5-trisphosphate; IP_3_R3, Inositol 1,4,5-trisphosphate receptor type 3; IRE1α, inositol-requiring enzyme 1 alpha; KOR, κ-opioid receptor; LHb, lateral habenula; LPS, lipopolysaccharide; LTD, long-term depression; LTP, long-term potentiation; MAMs, mitochondria-associated endoplasmic reticulum membranes; MAPK, mitogen-activated protein kinase; MAOIs, monoamine oxidase inhibitors; MBP, myelin basic protein; MCT1, monocarboxylate transporter 1; MCT2, monocarboxylate transporter 2; MCT4, monocarboxylate transporter 4; MDD, major depressive disorder; mGluRs, metabotropic glutamate receptors; MOR, μ-opioid receptor; mPFC, medial prefrontal cortex; mRNA, messenger RNA; MRS, magnetic resonance spectroscopy; mTOR, mammalian target of rapamycin; mTORC1, mammalian target of rapamycin complex 1; mTORC2, mammalian target of rapamycin complex 2; NE, norepinephrine; NF-κB, nuclear factor kappa B; NGF, nerve growth factor; NMDA, N-Methyl-D-aspartate; NMDAR, N-Methyl-D-aspartate receptor; NO, nitric oxide; NOP, nociceptin/orphanin FQ peptide receptor; NTX, naltrexone; OCD, obsessive-compulsive disorder; OPC, oligodendrocyte progenitor cell; PENK, proenkephalin; PERK, protein kinase R-like endoplasmic reticulum kinase; PET, positron emission tomography; PDYN, prodynorphin; PFC, prefrontal cortex; PI3K, phosphoinositide 3-kinase; PIP2, phosphatidylinositol 4,5-bisphosphate; PIP3, phosphatidylinositol 3,4,5-trisphosphate; PKA, protein kinase A; PKC, protein kinase C; PLC, phospholipase C; PNN, perineuronal net; PNOC, prepronociceptin; PNS, peripheral nervous system; POMC, proopiomelanocortin; PSD-95, postsynaptic density protein 95; PTSD, post-traumatic stress disorder; PV, parvalbumin; RNA, ribonucleic acid; ROS, reactive oxygen species; S1R, sigma-1 receptor; scRNA-seq, single-cell RNA sequencing; SNARE, soluble N-ethylmaleimide-sensitive factor attachment protein receptor; SNRIs, serotonin-norepinephrine reuptake inhibitors; SOCE, store-operated Ca^2+^ entry; SPT, sucrose preference test; SSRIs, selective serotonin reuptake inhibitors; SST, somatostatin; STIM1, stromal interaction molecule 1; TAMM41, translocator assembly and maintenance protein 41 homolog; TCAs, tricyclic antidepressants; TMD, transmembrane domain; TNF-α, tumor necrosis factor alpha; TRD, treatment-resistant depression; TRP, transient receptor potential; TSC2, tuberous sclerosis complex 2; TST, tail suspension test; TTX, tetrodotoxin; UPR, unfolded protein response; VEGF-A, vascular endothelial growth factor A; XBP1, X-box binding protein 1.
