# Supplementary material for: Integrating multi-receptor and neuron–glia mechanisms of ketamine's antidepressant action: a convergence framework for understanding efficacy
Source: Front Mol Neurosci. 2026 Oct 21;19:1950244. doi: 10.3389/fnmol.2026.1950244 (PMC13732179; doi:10.3389/fnmol.2026.1950244)
Supplement: Supplementary file 1 [file Table_1.docx]

Supplementary Table 1. Active clinical studies of ketamine were selected to represent depression, anxiety, PTSD, bipolar disorder, suicidal ideation, and OCD, focusing on currently active, large-enrollment, internationally diverse trials in which ketamine is a confirmed treatment arm.

| **Trial** | **Disorder** | **Contry** | **Start** | **End** | **Group of patients** | **Drug** |
| --- | --- | --- | --- | --- | --- | --- |
| **River at home ketamine protocols** | Depression; anxiety; ptsd; chronic pain | United states | 2023-10-01 | 2029-04-01 | Adults; n=50,000 | Ketalar, oral and nasal ketamine at home with no therapy, |
| **Retrospective analyses of the greenbrook database evaluating mental health treatments** | Depression; anxiety; ocd | United states | 2025-05-15 | 2035-12 | All ages; n=12,000 | Spravato (s-ketamine), nasal spray |
| **Intensified pharmacological treatment for schizophrenia, mdd and bipolar depression after first-time treatment failure** | Depression; bipolar disorder; psychosis | Israel | 2025-04-27 | 2028-06-30 | Adults; n=1,254 | Esketamine nasal product, esketamine hydrochloride, ketamine hydrochloride |
| **University of iowa interventional psychiatry service patient registry** | Depression; | United states | 2020-11-02 | 2050-08 | Adults; n=1,000 | Esketamine, ketamine |
| **Ketamine treatment plans for chronic conditions** | Depression; anxiety; chronic pain | United states | 2023-01-01 | 2029-06-01 | Adults; n=500 | Ketamin, ora lor nasal |
| **Esketamine combined with remimazolam on postoperative sleep disturbance and anxiety** | Anxiety | China | 2023-12-01 | 2024-12-10 | Adults; n=400 | Esketamine |
| **Esketamine nasal spray in real-world settings in treatment-resistant depression** | Depression; anxiety | Italy | 2022-11-01 | 2030-08 | Adults; n=100 | Esketamine, nasal |
| **In midazolam vs in dexmedetomidine vs in ketamine during minimal procedures in pediatric ed** | Anxiety | United states | 2023-11-14 | 2025-06 | Children; n=90 | Ketamine, nasal |
| **Enhancing week-long psychological treatment for ptsd with ketamine** | Ptsd | Israel; united states | 2023-08-21 | 2031-08-01 | Adults; n=162 | Ketamine |
| **Imaging sv2a in mood disorders** | Depression; ptsd | United states | 2016-04 | 2027-03 | Adults; n=130 | Ketamine |
| **Ketamine for pain, opioid use, and mental health in orthopedic trauma patients** | Depression; ptsd; chronic pain; substance use disorder | United states | 2025-11-06 | 2028-05 | Adults; n=90 | Ketamine |
| **Ampa receptor components of the anti-depressant ketamine response** | Depression; ptsd | United states | 2023-09-07 | 2032-12 | Adults; n=50 | Ketamine |
| **The impact of ampa receptor blockade on ketamine's anti-suicidal effects** | Depression; ptsd; bipolar disorder; suicidal ideation | United states | 2023-04-03 | 2033-03 | Adults; n=30 | Ketamine |
| **Ketamine associated acc gaba and glutamate change and depression remission** | Depression; bipolar disorder | United states | 2019-01-03 | 2026-12 | Adults; n=18 | Ketamine |
| **Chart review of patients undergoing ketamine infusions** | Depression; ptsd; bipolar disorder; ocd | Canada | 2019-12-03 | 2023-09-14 | Adults; n=891 | Ketamine hydrochloride |
| **Efficacy, safety and pk study of inhaled esketamine in treatment-resistant bipolar depression** | Depression; bipolar disorder | Poland | 2019-03-28 | 2021-02-19 | Adults; n=88 | Esketamine dpi - low dose, esketamine dpi - medium dose, drug: esketamine dpi - high dose |
| **Ketamine pk/pd for postpartum depression and pain after cesarean delivery** | Depression; chronic pain | United states | 2025-03-28 | 2028-03 | Adults; n=50 | Ketamine, ketalar |
| **Ketamine-assisted integrative treatment for veterans with chronic low back pain and comorbid depression** | Depression; chronic pain | United states | 2025-12-03 | 2029-06-30 | Adults; n=44 | Ketamine hydrochloride |
| **The effect of outpatient ketamine infusion on chronic neuropathic pain and ptsd** | Ptsd; chronic pain | United states | 2024-10-02 | 2027-05 | Adults; n=30 | Ketamine |
| **Neurobiology of suicide** | Depression; suicidal ideation | United states | 2015-12-01 | 2030-07-21 | Adults; n=325 | Ketamine hydrochloride |
| **I-cbt and iv ketamine for suicidality in treatment-resistant depression (rct)** | Depression; suicidal ideation | Canada | 2024-01-02 | 2026-06 | Adults; n=110 | Ketamine hydrochloride |
| **Long-term maintenance with ketamine and esketamine for reduction of suicide in high-risk patients** | Depression; suicidal ideation | United states | 2022-10-13 | 2026-06-01 | Adults; n=100 | Ketamine, esketamine |
| **Antidepressant effects of (2r,6r)-hnk, an enhancer of synaptic glutamate release, in trd** | Depression; suicidal ideation | United states | 2024-11-06 | 2027-07-01 | Adults; n=50 | (2r,6r)-hydroxynorketamine |
| **Examining mu opioid mechanisms of ketamine's rapid effects in ocd (mket2)** | Ocd; substance use disorder | United states | 2024-02-24 | 2028-11 | Adults; n=150 | Ketamine |
| **Ketamine in ocd: efficacy and effects on stress and cognition** | Ocd | Austria | 2022-10-01 | 2026-06 | Adults; n=60 | Ketamine |
| **Pilot study of rr-hnk in ocd** | Ocd | United States | 2026-01-23 | 2029-11-30 | Adults; n=45 | Rr-hnk/hydroxynorketamine |
| **Understanding how ketamine brings about rapid improvement in ocd** | Ocd | United States | 2016-06 | 2026-06 | Adults; n=120 | Ketamine |
| **Ketamine alcohol (in treatment-resistant depression)** | Depression; substance use disorder | United States | 2014-04-23 | 2028-09 | Adults; n=60 | Ketamine |
| **Act for alcohol use disorder and depression** | Depression; substance use disorder | Canada | 2025-05-01 | 2028-06-01 | Adults; n=30 | Ketamine |
| **Ketamine for combined depression and alcohol use disorder** | Depression; substance use disorder | Not specified | 2025-01-01 | 2027-07-01 | Adults; n=34 | Ketamine hydrochloride |
| **Ketamine-enhanced therapy for alcohol use disorder and depression (ket-dual)** | Depression; substance use disorder | Australia | 2025-12-10 | 2027-12-10 | Adults; n=20 | Ketamine |
| **Ketamine-enhanced therapy for alcohol use disorder and depression (ket-dual)** | Depression; substance use disorder | Australia | 46001 | 2027-12-10 | Adults; n=20 | Ketamine |
